# EpCAM supports exit from pluripotency of embryonic stem cells via Eomes

**DOI:** 10.1038/s41419-026-08734-w

**Published:** 2026-04-11

**Authors:** Ningyue Gong, Mahesh Gouda, Ana Marija Balaz, Jiahang Song, Gisela Kranz, Julia Hess, Philipp Baumeister, Kristian Unger, Vera Katalina, Martin Canis, Olivier Gires

**Affiliations:** 1 Department of Otorhinolaryngology, LMU University Hospital, LMU, Munich, Germany; 2 Department of Psychiatry and Psychotherapy University Hospital Bonn, University of Bonn Venusberg-Campus 1, Bonn, Germany; 3 Research Unit Translational Metabolic Oncology (TMO), Institute for Diabetes and Cancer (IDC), Helmholtz Diabetes Center, Helmholtz Munich, Neuherberg, Munich, Germany; 4 Department of Radiation Oncology, LMU University Hospital, Ludwig Maximilians University Munich, Munich, Germany; 5 Joint Heidelberg-IDC Translational Diabetes Program, Department of Inner Medicine I, Heidelberg University Hospital, Heidelberg, Germany; 6 German Center for Diabetes Research (DZD), Neuherberg, Germany; 7 Bavarian Cancer Research Center (BZKF), Munich, Germany; 8 German Cancer Consortium (DKTK), Partner Site, Munich, Germany; 9 Comprehensive Cancer Center (CCC), Munich, Germany

**Keywords:** Cell signalling, Differentiation

## Abstract

Epithelial cell adhesion molecule (EpCAM) is a tumor-associated antigen that marks pluripotent embryonic stem cells (ESCs). Regulation of *Epcam* expression yields a spatiotemporal patterning during embryogenesis that is thoroughly mimicked in a 3D model of spontaneous differentiation of embryoid bodies (EBs). Here, we present a role of EpCAM in exit from pluripotency of murine ESCs (mESCs) to establish cardiomyocytes in EBs. Comparative transcriptomic analysis of wildtype and *Epcam*-knockout mESCs at strategic time points of spontaneous differentiation uncovered molecular deficiencies of *Epcam*-knockout ESCs in “Wnt signaling” and “Heart development”. Multi-level bioinformatic analyses revealed central lineage-defining transcription factors *Eomes*, *Foxa2*, and *Gata6* as differentially expressed genes (DEGs) that are misregulated in *Epcam*-knockout mESCs. Gene expression association of *Epcam* with *Eomes*, *Foxa2*, and *Gata6* was prominent at day three of spontaneous differentiation, representing primitive streak formation in EBs. Interrogation of public single-cell RNA sequencing (scRNAseq) datasets supported a co-expression of *Epcam* and *Eomes* at early stages of murine embryogenesis in epiblast, primitive streak, nascent mesoderm, extraembryonic ectoderm and endoderm. Newly generated scRNAseq of wildtype mESCs in spontaneous differentiation delineated the formation of epiblast, primitive streak, endo- and mesoderm cells, and cardiomyocytes. Expression and pseudotime analysis positioned *Epcam* expression slightly ahead of *Eomes* at the transition of early to late primitive streak, along with rising Wnt signaling. Accordingly, conditional re-expression of *Epcam* or *Eomes* but not of *Foxa2* or *Gata6* complemented differentiation defects of *Epcam*-knockouts and confirmed an involvement of Wnt signaling in the EpCAM-dependent activation of *Eomes*. Hence, defective exit of pluripotency in *Epcam*-deficient ESCs is linked to *Eomes* regulation via Wnt signaling.

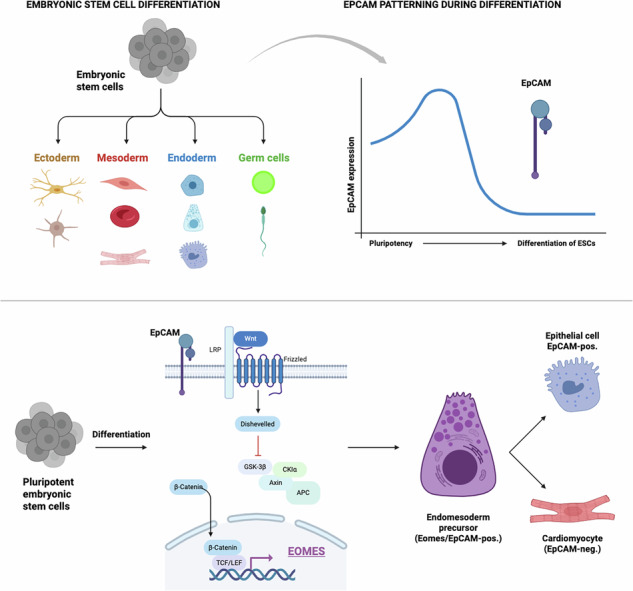

## Introduction

Murine embryonic stem cells (mESCs) are self-renewing and pluripotent cells that give rise to undifferentiated progeny upon symmetric division and to ecto-, meso-, and endodermal lineages following asymmetrical division [1–3]. Central aspects of embryonic development can be thoroughly recapitulated in a manageable in vitro 3D model of embryoid bodies (EBs) [4]. Murine EBs have facilitated the identification of transcriptomic changes associated with early and late pluripotency, exit from pluripotency, primitive streak formation, and cell specification within a time frame of five days [5]. Spontaneous differentiation of EBs in the absence of any stimulant gives rise to various cell lineages, prominently mesoderm-derived cardiomyocytes [6, 7].

Epithelial cell adhesion molecule EpCAM is a pan-carcinoma antigen [8]. that is also expressed on the cell surface of human and murine pluripotent stem cells, in tumor-initiating cells of various carcinoma entities [9–11]. and in liver progenitors including hepatic endoderm [12–14]. Although EpCAM is commonly regarded as an epithelial marker, EpCAM´s expression is substantially more intricate, and programmed patterning occurs along different cell lineages. Single-cell level analysis of the expression of *Epcam* during murine gastrulation uncovered a complex patterning during differentiation. Strict loss of *Epcam* gene expression was observed in nascent mesoderm progenitors whereas endodermal lineages maintained high levels of *Epcam*. Patterning of EpCAM was confirmed in perinatal mouse embryos, with a selective expression in cells of the endodermal lineage [14, 15]. Loss- and gain-of-function of *Epcam* in mESCs demonstrated a requirement for a strictly regulated expression across lineages, as over-expression and knockout of *Epcam* resulted in faulty differentiation [15].

An involvement of *Epcam* in the regulation of pluripotency and differentiation was likewise reported for human ESC (hESC) and induced pluripotent stem cell (iPSC) [16–19]. ScRNAseq analysis of differentiating human iPSC revealed a predominant expression of *Epcam* in pluripotent iPSC, early and late primitive streak, and in endodermal derivatives. Pseudotime analysis depicted a fluctuating expression of *Epcam* starting with a pronounced presence in pluripotent cells and primitive streak. In the pseudotime stream, *Epcam* expression was substantially reduced in mesodermal progenitors, early cardiac progenitors, and definitive endoderm. Further progression into mesodermal, cardiac, and endothelial lineages was characterized by complete loss of *Epcam*, whereas definitive and hepatic endoderm cells experienced a strong enhancement of *Epcam* expression to levels superior to pluripotent iPSC [14]. In hESC, the intracellular domain EpICD of EpCAM, which is generated *via* regulated intramembrane proteolysis (RIP) of full-length EpCAM [19–21]. binds and regulates promoters of pluripotency factors Oct3/4, Nanog, Sox, KLF4, and cMyc [19]. The initial step of RIP of EpCAM sheds a soluble extracellular domain EpEX that promotes the transcription of pluripotency genes upon activation of the epidermal growth factor receptor (EGFR), signal transducer and activator of transcription 3 (STAT3), and LIN28 [17]. In colorectal [22]. and head and neck squamous cell carcinoma (HNSCC) [23, 24]. EpEX functions as an EGFR ligand modulating epithelial-to-mesenchymal transition (EMT). During mesodermal differentiation of mESC and after EMT induction in carcinoma cells, endocytosis and degradation of EpCAM support its gradual loss at the plasma membrane [25]. Thus, evidence suggests regulatory functions of EpCAM in stem and carcinoma cell differentiation, but molecular networks and key regulatory genes remain insufficiently characterized [26]. Here, EpCAM´s role in mESC differentiation was interrogated through scRNAseq analysis and a comparative, temporal transcriptomic analysis of wild-type and knockout cell lines. We reveal an association of EpCAM with cardinal lineage-regulatory transcription factors Eomesodermin (*Eomes*), Forkhead box A2 (*Foxa2*), and GATA-binding factor 6 (*Gata6*), and identify *Eomes* as important regulator of EpCAM-dependent cardiomyocyte formation.

## Results

### Kinetic changes associated with loss of EpCAM in mESC

**Fig. 1:**
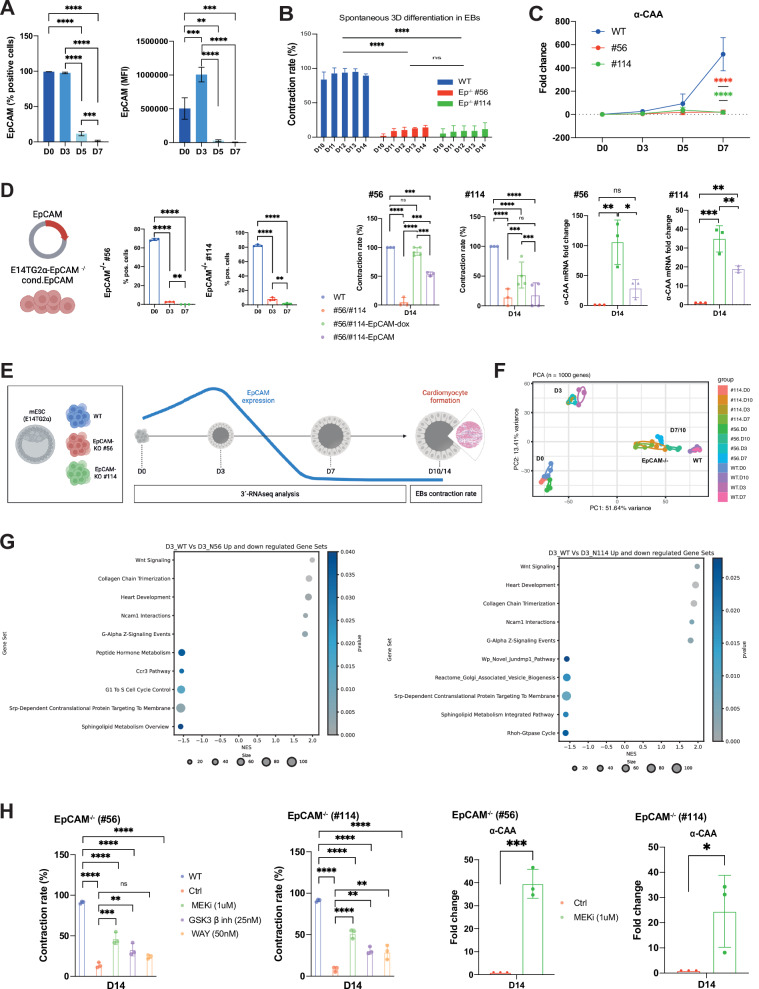
EpCAM expression and impact during spontaneous differentiation of ESCs. **A** EpCAM expression was analyzed in WT E14TG2α mESCs by flow cytometry during spontaneous differentiation in embryoid bodies (EBs). Percentages of EpCAM-positive cells (left) and normalized EpCAM mean fluorescence intensity (EpCAM MFI, right) with SD from *n* = 3 independent measurements are shown. **p-value < 0.01; *** < 0.001; **** < 0.0001. **B** WT and EpCAM^−/−^ cells were subjected to spontaneous differentiation in EBs in the absence of LIF. Contraction rates were quantified from D10 to D14 as mean with SD of *n* = 3 independent experiments. ****p-value < 0.0001; ns: not significant. **C** Cardiomyocyte marker α-CAA was quantified by qRT-PCR in WT and EpCAM^−/−^ E14TG2α over time. Shown are mean with SD of *n* = 3 independent experiments. ****p-value < 0.0001. **D** E14TG2α EpCAM^−/−^ ESCs (clones #56 and #114) were stably transfected with conditional, doxycyclin-responsive vectors expressing EpCAM in fusion with enhanced green fluorescence protein (EGFP). Transgene expression was induced via doxycycline in 2D-culture and EBs were generated. Upon doxycycline removal, transgene expression was assessed upon flow cytometry quantification of EpCAM-EGFP. Shown are mean with SD from *n* = 3 independent experiments. *p-value < 0.05, ** < 0.01, *** < 0.001, **** < 0.0001. Contraction rates of EBs from WT, EpCAM^−/−^ ESCs (clones #56 and #114), and EpCAM-EGFP re-expression clones at D14 of spontaneous differentiation are shown as mean with SD from n = 3 independent experiments including at least six EBs per experiment and treatment. Where indicated, EpCAM^−/−^ ESCs (#56 and #114) re-expression clones were pre-treated with doxycycline (dox). Expression of ^α-^CAA was quantified by qRT-PCR at day 14. Shown are mean with SD from *n* = 3 independent experiments performed in triplicates. *p-value < 0.05, ** < 0.01, *** < 0.001, **** < 0.0001. **E** Scheme of experimental procedure. Wild-type (WT) and *Epcam* knockout (EpCAM^−/−^) murine E14TG2α were cultured in hanging drops in the absence of LIF. Embryoid bodies (EBs) were cultured for 14 days, and contraction was quantified. At D0, 3, 7, and 10, 3´-bulk sequencing was performed on quadruplicates. **F** Principal component analysis (PCA) of WT and EpCAM^−/−^ cells (clones #56 and #114) at D0, D3, D7, and D10. **G** Top ten differential gene sets identified upon GSEA using within GSEA-MSigDB are depicted with normalized enrichment scores (NES) and normalized p-values. Shown are the GSEA from WT versus EpCAM^−/−^ cell clones #56 and #114 at D3. **H** Contraction rates of EBs from WT and EpCAM^−/−^ ESCs (clones #56 and #114) at D10 and D14 of spontaneous differentiation are shown as mean with SD from *n* = 3 independent experiments including at least six EBs per experiment and treatment. Where indicated, EpCAM^−/−^ ESCs (clones #56 and #114) were treated with MEK inhibitor (MEKi 1 µM), GSK3β (25 nM), or WAY (50 nM) (**C**). *p-value < 0.05, ** < 0.01, *** < 0.001, **** < 0.0001; ns: not significant. Expression of *α-Caa* was quantified by qRT-PCR at day 14 of spontaneous differentiation in the presence or absence of MEK inhibitor in EpCAM^−/−^ ESCs (clones #56 and #114). Shown are mean with SD from *n* = 3 independent experiments performed in triplicates. *p-value < 0.05, ** < 0.01, *** < 0.001.

Cell surface expression of EpCAM was analyzed over time during spontaneous differentiation of mESC line E14TG2α in EBs. Under pluripotency conditions, > 98% of cells expressed EpCAM to high levels. At D3 of differentiation, EpCAM expression was homogeneously enhanced and gradually declined at D5 and D7 to less than 10% positive cells (Fig. 1A and Supplementary Fig. 1A, B). EpCAM was selectively expressed in EBs upon spontaneous differentiation and was mutually exclusive to cardiomyocyte marker alpha cardiac actin (α-CAA) (Supplementary Fig. 1C).

CRISPR-Cas9-generated EpCAM^−/−^ single cell clones [15]. Expressed no detectable levels of EpCAM (Supplementary Fig. 1D, E) and were impaired in spontaneously forming contracting cardiomyocytes (Fig. 1B). This impairment was accompanied by a loss of α-CAA mRNA induction in knockout clones compared to WT cells (Fig. 1C) and reduced levels of α-CAA protein without recognizable patterning (Supplementary Fig. 1F). The phenotype specificity was confirmed upon conditional re-expression of EpCAM-EGFP from a doxycycline-inducible promoter in *Epcam*^−/−^ clones #56 and #114. Expression of EpCAM-EGFP was inducible and resulted in correct sub-cellular localization at the plasma membrane (Supplementary Fig. 2A, B). Expression analysis further revealed leakage in clone #56 in the absence of doxycycline. Leakage in clone #56 led to a 2.26-fold enhanced EpCAM-EGFP expression compared to clone #114. Doxycycline treatment induced a strong EpCAM-EGFP expression in 91.2% and 88.7% of #56 and #114 cells, respectively (Supplementary Fig. 2C, D). Transgene expression was induced before EB formation and relieved during spontaneous differentiation to mimic EpCAM´s gradual loss along differentiation in WT ESCs (Fig. 1D). In knockout clone #56, EpCAM leakage in absence of doxycycline at the time point of EB formation complemented cardiomyocyte formation to 50% of WT. Enhanced expression of EpCAM upon doxycycline addition restored WT contraction rates in clone #56 at D14 ( > 90% of WT). Weak EpCAM leakage in clone #114 had no influence on contraction rates in absence of doxycycline, whereas EpCAM re-expression restored contraction to 50% of WT after doxycycline addition. α-CAA expression levels confirmed contraction rates observed after EpCAM re-expression in EpCAM^−/−^ clones (Fig. 1D). Thus, enhanced expression of EpCAM at early stages of spontaneous differentiation revealed essential to ensure cardiomyocyte formation in EBs.

Next, EpCAM´s impact on the transcriptome was addressed using bulk 3´-RNA sequencing (3´-RNAseq) across cardinal stages of spontaneous differentiation (GSE293121). Pluripotent mESCs (D0; in the presence of LIF) and spontaneously differentiating EBs at D3 of LIF withdrawal corresponding to genetic changes in association with primitive streak, and D7 and D10 representing early and late time points of differentiation were assessed for WT and knockout mESC lines (Fig. 1E). Principal component analysis (PCA; *n* = 10,000 most variant genes) showed clustering of biological replicates for each cell lines and time points (Fig. 1F). Differentially expressed genes (DEGs; log2FC > 1, FDR < 0.05) consistently observed between WT and both EpCAM^−/−^ clones revealed minor transcriptomic differences under pluripotency (D0, *n* = 36 DEGs). At D3, D7, and D10, *n* = 58, *n* = 448, and *n* = 309 DEGs were identified, respectively (Supplementary Fig. 3A and Supplementary Table 1). At D3, most DEGs were down-regulated in EpCAM^−/−^ cells versus WT, whereas up- and down-regulated DEGs were distributed more evenly at D7 and D10. Hence, kinetic transcriptomic differences between WT and EpCAM^−/−^ ESCs suggested early dysregulation starting at D3 and amplifying at D7 and D10.

Gene set enrichment analysis (GSEA) using the Molecular Signature Database (MSigDB) delivered insights in murine canonical pathways affected by EpCAM. Both EpCAM^−/−^ clones used in this study were highly similar regarding transcriptional differences compared to WT cells with *n* = 117 common gene sets (Supplementary Fig. 3B, C). Starting from D3 and ongoing, WT cells were characterized by an up-regulation of the canonical pathways (CP) “Wnt signaling”, “Heart development”, and “NCAM1 interactions” compared to EpCAM^−/−^ cell clones (Fig. 1G and Supplementary Fig. 3D). Common down-regulated hallmarks in WT versus EpCAM^−/−^ were “Golgi associated vesicle biogenesis” (D0 and D7), “SRP-dependent cotranslational protein targeting to membrane” (D3), and “Mitochondrial translation” (D10). Hence, differences in regulated gene sets between WT and EpCAM^−/−^ cells mirrored functional deficiencies of knockout cells in “Heart development” and indicated “Wnt signaling” as potentially involved.

Treatment with Wnt-activating compounds AZD6244 (MEK inhibitor [27, 28]. CHIR99021 (GSK3β inhibitor), and WAY262611 restored contraction rates in EpCAM^−/−^ clones. AZD6244 had the strongest restoring effect and rescued contraction rates to 50.9% and 56.4% of WT cells in EpCAM^−/−^ clones #56 and #114, respectively. CHIR99021 and WAY262611 recovered 36.6%/33.8% and 26%/31.9% of the WT contraction rate in EpCAM^−/−^ clones (Fig. 1H). Based on the superior complementation of the EpCAM knockout phenotype by MEKi, we interrogated the levels of α-CAA following treatment and show a significant up-regulation (Fig. 1H). Notably, Wnt signaling was required in the early phase of EB differentiation from D0 to D6, whereas a permanent activation throughout the differentiation period was ineffective. Thus, EpCAM-related defects in exit from pluripotency and differentiation are associated with disturbance of Wnt activation in the early phase of ESC differentiation.

### Identification of functional DEGs

**Fig. 2:**
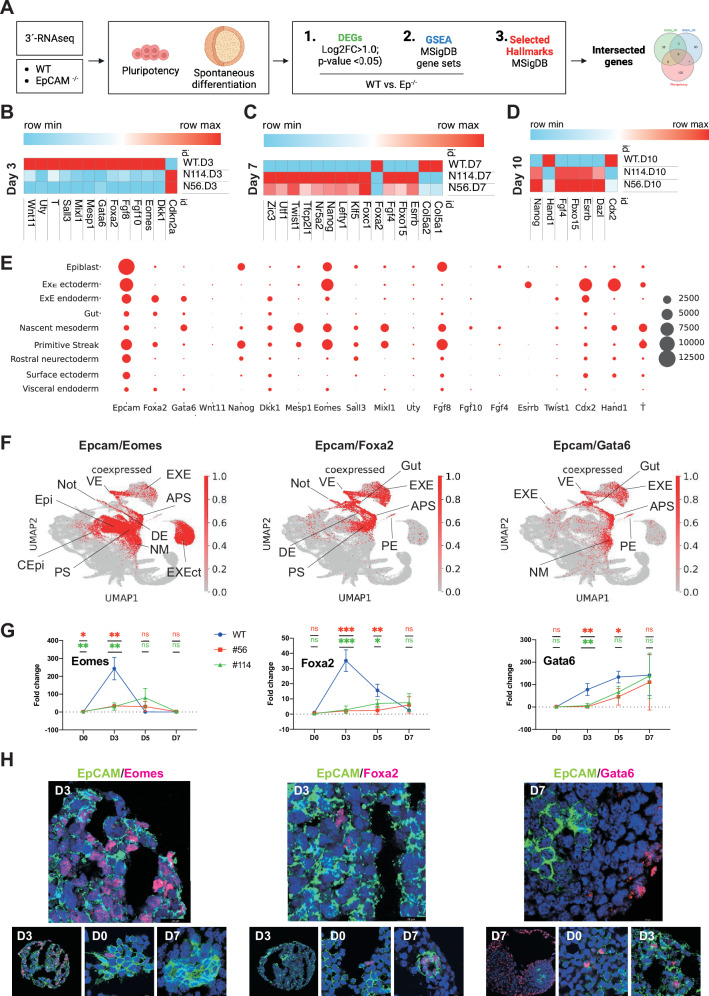
Definition of EpCAM-associated functional DEGs in mESC exit from pluripotency. **A** Schematic representation of the workflow and three-tiered selection applied to identify functional DEGs (fDEGs) between WT and EpCAM^−/−^ cells. **B**–**D** Heatmaps of functional DEGs at D3, D7, D10 of spontaneous differentiation identified between WT and EpCAM^−/−^ cells (clones #56 and #114). **E** Expression of *Epcam* and *n* = 18 fDEGs is shown as dot plot graph in the indicated murine lineages. **F** UMAP representation of co-expression patterns of *Epcam* with *Eomes*, *Foxa2*, and *Gata6*, respectively, in lineages of murine development. APS anterior primitive streak, DE definitive endoderm, EXE extraembryonic endoderm, EXEct: extraembryonic ectoderm, Epi epiblast, G gut, IM intermediate mesoderm, MM mixed mesoderm, NM nascent mesoderm, NC notochord, PE parietal endoderm, PS primitive streak, VE visceral endoderm. **G** WT and EpCAM^−/−^ ESCs (clones #56 and #114) were subjected to spontaneous differentiation in EBs and were analyzed by qRT-PCR at indicated time points for the expression of *Eomes*, *Foxa2*, and *Gata6* in independent experiments (*n* = 3). Shown are mean with SD. *p-value < 0.05, ** < 0.01, *** < 0.001, **** < 0.0001; ns: not significant. Expression levels are displayed for WT (blue), EpCAM^−/−^ clones #56 (red) and #114 (green). **H** Immunofluorescence staining of EpCAM in combination with Eomes, Foxa2, and Gata6 is shown in wildtype E14TG2α cells at the indicated time points of spontaneous differentiation in EBs. Shown are representative sections of *n* = 3 independent experiments performed with multiple EBs.

Next, we sought to identify genes involved in EpCAM-dependent processes of mESC differentiation with particular focus on cardiomyocyte development, which we termed functional DEGs (fDEGs). For this purpose, a three-tiered selection was conducted on our bulk transcriptomic dataset. Tier 1: DEGs with a Log 2FC > 1.0 and FDR < 0.05 were identified between WT and both KOs. Tier 2: Significantly activated or suppressed hallmarks of the molecular signature database (MSigDB) were defined by GSEA comparing WT cells with both KO clones. Tier 3: DEGs from Tier 1 that contribute to selected hallmarks relevant to cardiomyocyte formation were extracted independently of significant changes in the corresponding hallmarks. Here we concentrated on “Pluripotency”, “Stem cell differentiation”, “Mesoendodermal differentiation”, “Mesoderm differentiation”, “Endoderm differentiation”, “Ectoderm differentiation”, “Heart development”, “Cardiac contraction”, and “WNT signaling”. Functional DEGs are the intersection of genes across tiers, ensuring they represent DEGs contributing to gene networks relevant to the phenotype of EpCAM knockouts (see scheme in Fig. 2A).

Under pluripotency conditions, central regulators of “Stem cell differentiation” and germ layer definition such as *Gata6*, *Nanog*, *Stat3*, *Esrrb*, and *Foxa2* were determined in the GSEA/hallmark intersect (Supplementary Fig. 4A). Expression of coding and non-coding transcripts related to primitive streak formation have been reported at D3 of spontaneous differentiation in EBs [5]. Shortly thereafter (D3.5) initiation of patterning of EpCAM expression occurs, which was associated with a complete loss of expression in mesodermal cells and retention in endodermal cells [15]. At D3, transcription factor (TF) *Foxa2* was determined as fDEG enriched in “Mesoendodermal differentiation” and “Heart development” (triple-intersected). TF *Gata6* was a triple-intersected fDEG identified in “Pluripotency”, “Stem cell differentiation”, “Endoderm differentiation”, and “Heart development” (Fig. 2B and Supplementary Fig. 4B). *Foxa2*, *Gata6*, and *Wnt11* were DEGs identified in the GSEA, and *Mesp1*, a central regulator of cardiomyocyte formation, and *DKK1*, a negative regulator of Wnt signaling, were identified as DEGs involved in “Mesoderm differentiation” and “Endoderm differentiation” (double-intersected). Further prominent regulators of differentiation included *T* (*Brachyury*), *Eomes*, and *Mixl1* (Fig. 2B and Supplementary Fig. 4B). Specifically assessing “Heart development” and “Cardiac contraction” hallmarks at this early time point of spontaneous differentiation, fibroblast growth factor 8 and 10 (*Fgf8*, *Fgf10*) and the ubiquitously transcribed tetratricopeptide repeat containing, Y-linked protein *Uty* were identified as DEGs. All DEGs at D3 were repressed in EpCAM^−/−^ clones except cyclin-dependent kinase inhibitor *Cdkn2a*, suggesting a lack of induction of central lineage regulators in combination with an induction of cell cycle inhibitors in EpCAM KOs (Fig. 2B and Supplementary Fig. 4B).

At D7 and D10, a total of *n* = 15 and *n* = 7 triple-intersected genes, respectively, were identified; four of which were shared between both differentiation time points, i.e*., Esrrb*, *Fbxo15*, *Fgf4*, and *Nanog*. fDEGs included pluripotency genes and central regulators of differentiation such as *Nanog*, *Twist1*, *Foxa2*, and *Klf5*. Collagen V alpha sub-units *Col5a1*, *Col5a2*, the endodermal switch *Foxa2* at D7, caudal-type homeobox protein 2 (*Cdx2*) and heart- and neural crest derivatives-expressed protein 1 (*Hand1*) at D10, were enhanced in WT cells, whereas all other functional DEGs showed an enhanced expression in both EpCAM^−/−^ clones (Fig. 2C, D and Supplementary Fig. 4C, D). Cdx2 is a TF expressed in intestinal epithelial cells that is relevant to the differentiation and maintenance of intestinal lining. Hand1 is a TF with an ill-understood role in cardiac morphogenesis. Thus, late time points of spontaneous differentiation in the absence of EpCAM were characterized by a reduced expression of TFs *Foxa2*, *Cdx2*, and *Hand1*, and the retention of pluripotency factors (*Nanog*, *Klf5*), indicating a defect in pluripotency exit.

### Single cell resolution of EpCAM-associated DEG expression in embryogenesis

Temporal and cell-specific expression of *Epcam* and associated fDEGs was investigated in murine embryogenesis in public scRNA-seq datasets covering post-gestation days E3.5-E6.75 and E6.5-E8.5, respectively [29, 30]. This approach was chosen to interrogate physiological relevance and co-expression patterns of EpCAM and fDEGs at single-cell resolution during murine in vivo embryogenesis.

At early pre-gastrulation time point E3.5, *Epcam* was strongly expressed, showed transient decrease at E4.5, and reinforced expression at E5.5, E6.5, and E6.75 (Supplementary Fig. 5A). *Eomes* was expressed in few cells at early time points E3.5-5.5 and was more strongly and frequently expressed in cells at days E6.5 and E6.75. *Foxa2* was not expressed at E3.5 and E5.5, showed a weak expression at E4.5, and strongest expression at E6.5/E6.75. *Gata6* was expressed in selected cells at E3.5 and E4.5, and very weakly at E6.5 (Supplementary Fig. 5A). Correlations of expression of fDEGs with *Epcam* are depicted in Supplementary Fig. 5B. Pluripotent and epiblast cells were mapped to gestation days and the expression of *Epcam* and functional DEGs was analyzed. *Epcam* was expressed in pluripotent cells, most strongly at E3.5, and in the epiblast at E5.5 and 6.5, and showed considerable overlap with *Eomes*, which was additionally expressed at E4.5 in pluripotent cells. *Foxa2* was primarily expressed in pluripotent cells at E4.5, in pluripotent and epiblast cells E6.5, and showed overlap with *Epcam*. *Gata6* was expressed in pluripotent cells at E3.5, E4.5, and E6.5, showing overlap with *Epcam* (Supplementary Fig. 5C).

The expression of *Epcam* and fDEGs was analyzed at later gestation days E6.5–E8.5 [29] (Supplementary Fig. 6A for detailed expression profiles and kinetics). Numbers of cells expressing *Epcam*, *Eomes*, *Fgf8*, and *T* showed a sharp increase at gestation day E7.5, starting as co-expression of *Epcam* with *Eomes* and *Fgf8* at E7.0–7.25 (Supplementary Fig. 6B-D). *Epcam* was most frequently expressed in the epiblast, extraembyonic ecto- and endoderm, primitive streak, rostral neuroectoderm, and in fewer cells of the gut, nascent mesoderm, surface ectodem, and visceral endoderm (Fig. 2E). Co-expression of *Epcam* with *Eomes*, *Foxa2*, and/or *Gata6* was predominant in (anterior) primitive streak, and endodermal tissue. Specifically, co-expression of *Epcam* with *Eomes* occurred in the epiblast, (anterior) primitive streak, extraembryonic ectoderm, nascent mesoderm, extraembryonic ectoderm, and visceral endoderm. (Fig. 2F). This timely association of *Epcam* expression with Eomes, Foxa2, and Gata6 (Fig. 2G), and with Dkk1, Fgf8, Fgf10, Mesp1, Mixl1, T, and Wnt11 was confirmed at D3 of spontaneous differentiation in EBs of WT but not of EpCAM^−/−^ clones (Supplementary Fig. 7). Immunofluorescence staining of WT cells demonstrated a co-expression of EpCAM with Eomes and Foxa2 in single cells at D3 and a selective Gata6 expression in the absence of EpCAM at D7 (Fig. 2H).

To decipher cross-species associations of *Epcam* with central genes in embryonic differentiation, we assessed expression patterns in the landscape of human gastrulation in the public dataset GSE155121 [31]. Carnegie stages 12-16 of human embryonic development, representing post-fertilization age 29-31 days to day 39, were examined for the expression of fDEGs (Supplementary Fig. 8A). *Epcam* expression was observed at CS12, CS13-14, and CS15-16, and primarily in epithelium, epidermis, and endoderm. Except for *Fgf4*, *ESRRB*, *Nanog*, and *TBXT*, which were expressed in only very few cells, all other fDEGs showed spatiotemporally defined expression patterns (Supplementary Fig. 8A). *Epcam* was co-expressed with *Foxa2*, *Gata6*, and *Dkk1* in single endodermal cells. In the epidermis, co-expression was seen with *Stat3*, *Fgf8*, and *Twist1*, whereas neural progenitors co-expressed *Epcam* and *Foxa2*, *Sall3*, *Fgf8*, and *Twist1*. In somites and spanchnic lateral plate mesoderm (LPM), *Epcam* was co-expressed with *Gata6*, *Dkk1*, *Sall3*, and *Twist1* (Supplementary Fig. 8B). Similarly to murine embryonic development, *Epcam* was expressed in high numbers of single cells (Supplementary Fig. 8C), and strongest co-expression at the level of single cell numbers was seen with *Gata6*, *Foxa2*, *Dkk1*, and *Fgf8* (Supplementary Fig. 8D, E). Co-expression with *Eomes* could not be addressed based on low numbers of positive cells at these developmental stages. Co-expression of *Epcam* and *Foxa2* occurred in epidermis, epithelium, endoderm, and in few neural (progenitor) single cells. Co-expression with *Gata6* was less confined and observed in splanchnic LPM, endoderm, and endothelium. The expression of *Epcam*, *Foxa2*, and *Gata6* occurred specifically in epidermis, epithelium, and endoderm. Furthermore, co-expression of *Epcam* with *Dkk1*, *Stat3*, and *Fgf8* was seen in endoderm, epidermis, epithelium, and neural cells (Supplementary Fig. 8F).

### scRNAseq analysis of mESC in spontaneous differentiation

**Fig. 3:**
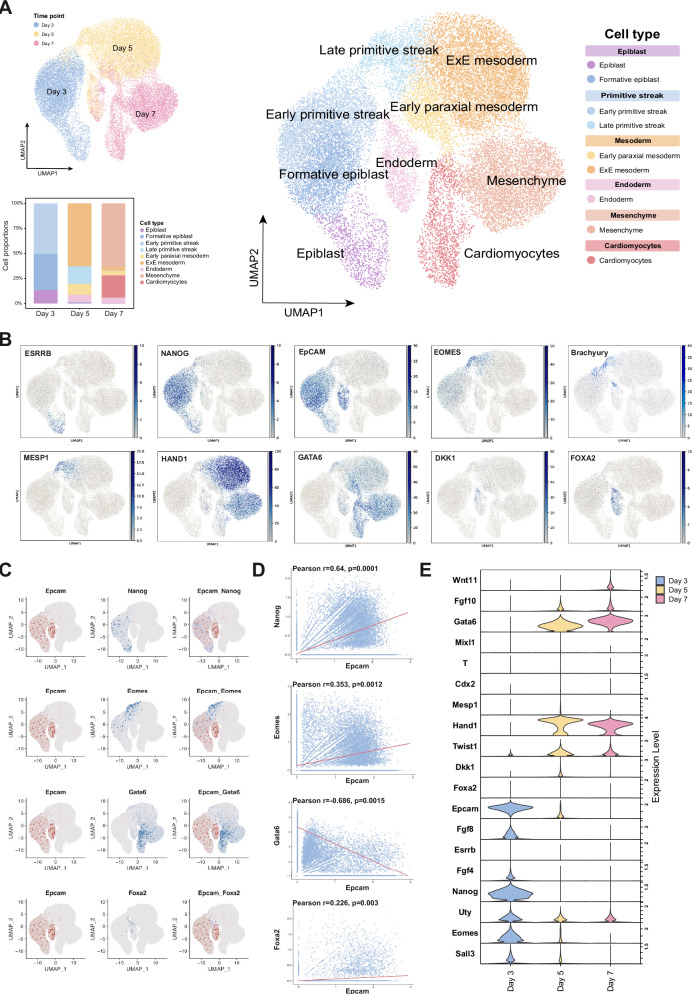
scRNAseq analysis of mESC in spontaneous differentiation. **A** WT E14TG2α mESCs were subjected to spontaneous differentiation in EBs for three, five, and seven days and were analyzed by scRNAseq. Shown are uniform manifold approximation and projections (UMAP) of the differentiation days (upper left) and cell types (right) with proportions of cell types over time (lowe left). **B** UMAPs of *Epcam* and the indicated functional DEGs expressed over time in WT E14TG2α mESCs. **C**, **D** Co-expression of Epcam with Nanog, Eomes, Gata6, and Foxa2 is depicted at single cell-level in UMAPs (**C**) and in correlation plots with Pearson correlation and *p*-values (**D**). **E** Expression levels of *Epcam* and fDEGs (*n* = 18) is shown in violin plots over time (D3, 5, and 7).

Next, we resolved gene (co)-expression patterns at single cell-level during spontaneous differentiation of E14TG2α EBs. scRNAseq analysis yielded transcriptomes from *n* = 8,594, *n* = 11,243, and *n* = 8,248 cells at D3, 5, and 7, respectively (GSE318465). Gene expression-based clustering of single cells revealed segregated clusters at D3, D5, and D7, with increasing sub-clustering over time (Fig. 3A). Marker gene-based cell annotation defined epiblast, formative epiblast, and early primitive streak at D3, late primitive streak, extraembryonic mesoderm, early paraxial mesoderm, and endoderm at D5, and mesenchyme, endoderm, and cardiomyocytes at D7 (Fig. 3A and Supplementary Fig. 9A).

*Epcam* was expressed in the epiblast, formative epiblast, early primitive streak, late primitive streak, and endoderm. fDEG estrogen-related receptor beta (*Esrrb*) encodes a self-renewal and pluripotency-associated nuclear receptor co-expressed with *Epcam* in epiblast cells. *Epcam* was further co-expressed with pluripotency gene *Nanog* in epiblast, formative epiblast, early primitive streak and selected late primitive streak cells. In the latter, *Epcam* and *Nanog* were co-detected with *Brachyury*, *Mesp1*, and *Gata6*. Upon further differentiation to mesodermal cells, *Brachyury* and *Mesp1* were silenced, while *Hand1* was strongly induced. The expression of *Epcam* in endodermal cells was accompanied by Wnt signaling inhibitor *Dkk1* in selected cells and by central endodermal reprogramming transcription factor *Foxa2* (Fig. 3B). Co-expression at single cell level was consistently observed between *Epcam*, *Nanog*, *Eomes*, and *Foxa2*, while *Epcam* and *Gata6* showed exclusive expression patterns (Fig. 3C, D). The expression of *Epcam* and all fDEGs across D3, 5, and 7 demonstrated further association of *Epcam* with *Fgf8*, *Fgf4*, *Nanog*, *Uty*, *Eomes*, and *Sall3* at D3. Later stages of differentiation (i.e. D5 and 7) were characterized by loss of *Epcam* and expression of *Gata6*, *Twist1*, and *Hand1* (Fig. 3E and Supplementary Fig. 9B, C).

**Fig. 4:**
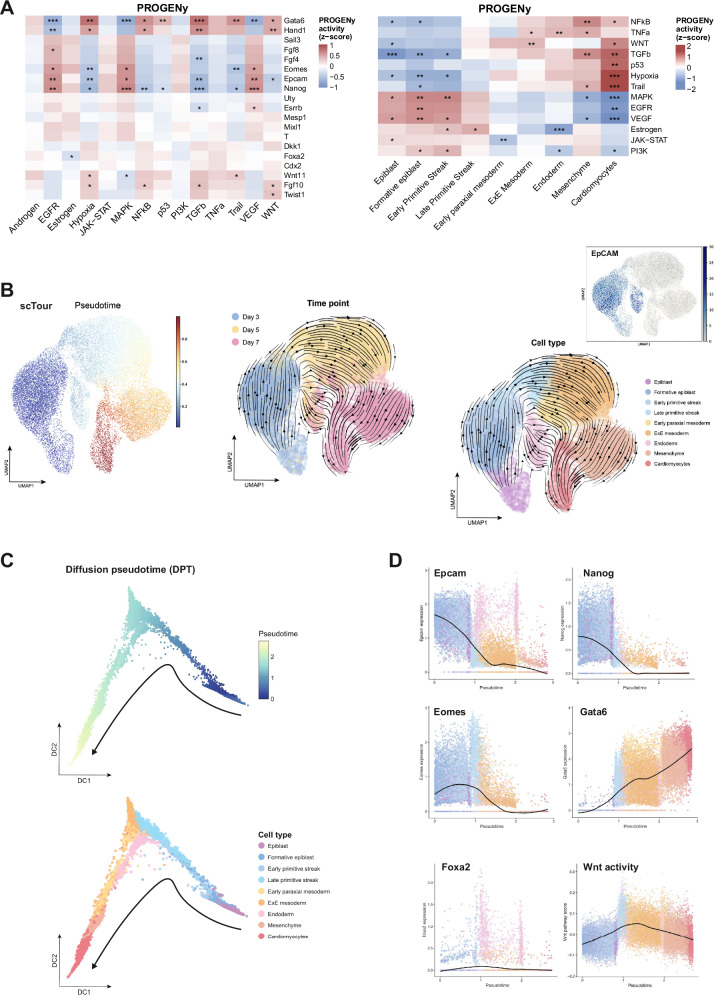
PROGENy and pseudotime analysis of mESC in spontaneous differentiation. **A** Correlation analysis of *Epcam* and fDEGs (*n* = 18) with pathway activities scores inferred using PROGENy at single cell-level are shown (left heatmap). Correlation analysis of cell types identified in spontaneously differentiating WT E14TG2α mESCs with pathway activities scores inferred using PROGENy at single cell-level are shown (right heatmap). **B**, **C** scTour- (**B**) and Diffusion pseudotime-based pseudotime analyses (**C**) of spontaneously differentiating WT E14TG2α mESCs are depicted over time and cell types (root cells: Epiblast). **D** Expression of Epcam, Nanog, Eomes, Gata6, and Foxa2, and Wnt signaling activity are plotted within the DPT-pseudotime stream of spontaneously differentiating WT E14TG2α mESCs. Color coding marks cell types as in (**C**).

Insights in signaling functions associated with fDEGs and cellular lineages were obtained upon correlation with PROGENy activity scores. *Epcam* and fDEGs expressed at early stage D3 were generally associated with stronger pathway activity scores than fDEGs expressed at later time points. *Epcam*, *Nanog*, and *Eomes* particularly strongly correlated with EGFR, MAPK, and VEGF signaling (Fig. 4A, left panel). These associations were corroborated by high positive correlations of *Epcam*/*Nanog*/*Eomes*^high^ cells of the epiblast and primitive streak with EGFR, MAPK, VEGF activities (Fig. 4A, right panel). Further differentiated cells of mesodermal and endodermal lineage were characterized by a sharp downregulation of these signaling pathways, and the induction of alternative pathways including NFκB, TNFα, TGFβ, p53, hypoxia, and Trail signaling. Increasing Wnt signaling was observed starting in late primitive streak cells and increased towards cardiomyocytes (Fig. 4A, right panel). Hence, *Epcam*, *Nanog*, and *Eomes* associated with elevated EGFR, MAPK, and VEGF activities in early mESC differentiation, and with the onset of Wnt signaling in late primitive streak.

Pseudotime trajectories of differentiating mESCs were inferred using scTour and Diffusion Pseudotime (DPT) with epiblast as root cells. While scTour infers time as a latent variable, DPT models diffusion-based cellular transitions from root cells [32, 33]. ScTour demonstrated the derivation of Epcam^high^ formative epiblast from epiblast cells, which further progressed into early and late primitive streak. Primitive streak cells separated to form *Epcam*^low/negative^ early paraxial and extraembryonic mesoderm, and *Epcam*^high^ endoderm. Mesodermal lineages progressed into mesenchyme and, ultimately, cardiomyocytes (Fig. 4B). DPT analysis confirmed the sequence of lineage formation and segregation of meso- and endodermal progeny at the primitive streak stage (Fig. 4C). Gene expression profiles along the DPT-based pseudotime stream revealed strong expression of *Epcam*, *Nanog*, and *Eomes* in epiblast and primitive streak, with elevated *Eomes* in late primitive streak that was accompanied by consistently increasing Wnt signaling. The latter signal was further enhanced in early mesodermal progenitors and decreased in more differentiated mesenchymal cells and cardiomyocytes, in which the expression of Gata6 was prominent (Fig. 4D).

Hence, scRNAseq analysis confirmed *Epcam* expression in early cells of spontaneously differentiating mESC and an overlapping pattern with *Eomes* and *Nanog* in epiblast and primitive streak. The data further suggest a critical function of *Epcam* during segregation of meso- and endodermal cells in the primitive streak, which is accompanied by increased Wnt signaling and sharp *Eomes* induction.

### Reconstitution of Eomes expression complements EpCAM deficiency

Defective cardiomyocyte formation in *Epcam* knockout mESCs may originate from a malformation of early primitive streak cells or a reduction of endodermal cells required for instructing cardiomyocytic mesodermal precursors [34]. Early co-expression of *Epcam* with *Eomes* and *Gata6* in late primitive streak and with *Foxa2* in endoderm prompted us to interrogate the capacity of these selected genes to rescue the phenotype of EpCAM^−/−^ clones. *Eomes*, *Gata6*, and *Foxa2* were individually re-expressed as EGFP fusions in EpCAM^−/−^ clones #56 and #114 using doxycycline-responsive expression vectors. Gene expression was induced before EB formation and relieved during spontaneous differentiation to mimic expression patterns observed in WT ESCs. Expression of all transgenes was inducible and resulted in correct sub-cellular localizations (Supplementary Fig. 10A, B). Quantification of EGFP-fusions by flow cytometry confirmed the inducibility of transgenes and revealed leakage of *Eomes*, and *Gata6* to varying degree, with clone #56 showing stronger *Eomes* leakage than clone #114 (Supplementary Fig. 11).

**Fig. 5:**
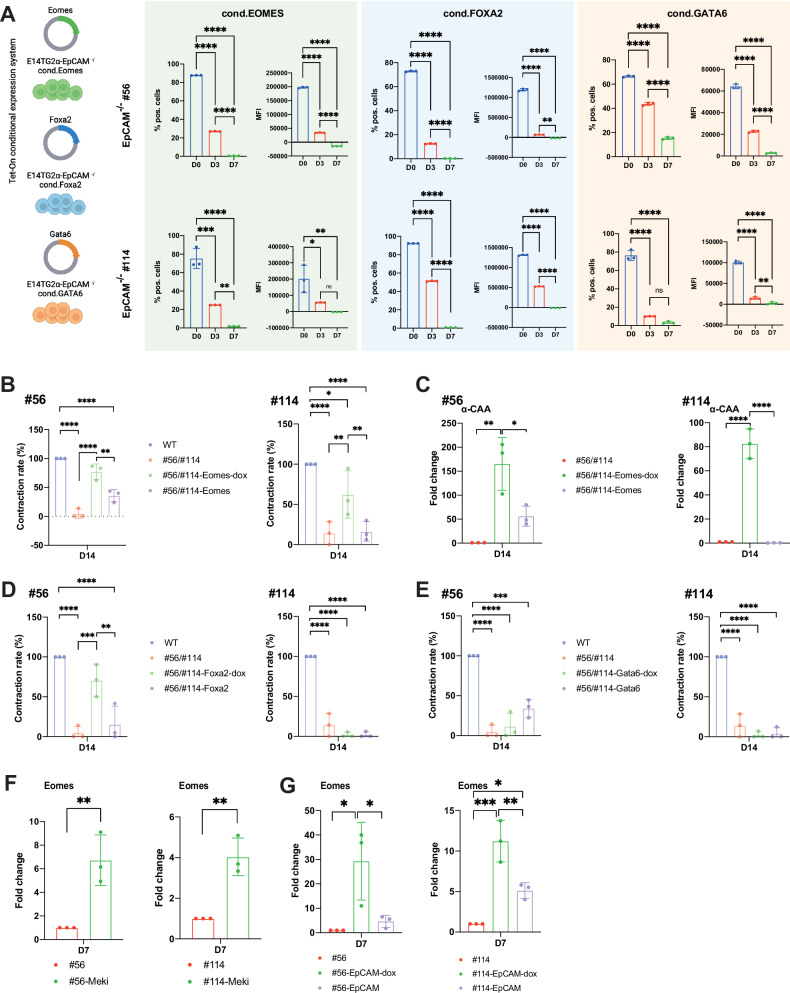
Temporal re-expression of *Eomes* complements knockout defects in 3D spontaneous differentiation. **A** E14TG2α EpCAM^−/−^ mESCs (clones #56 and #114) were stably transfected with conditional, doxycyclin-responsive vectors for *Eomes*, *Foxa2*, and *Gata6* in fusion with enhanced green fluorescence protein (EGFP). Transgene expression was induced via doxycyclin in 2D-culture and EBs were generated. Upon doxycycline removal, transgene expression was assessed upon flow cytometry quantification of EGFP. Shown are mean with SD from *n* = 3 independent experiments. *p-value < 0.05, ** < 0.01, *** < 0.001, **** < 0.0001. **B**, **D**, **E** Contraction rates of EBs from WT, EpCAM^−/−^ ESCs (clones #56 and #114), and Eomes (**B**), and Foxa2 (**D**) and Gata6 (**E**) re-expression clones at D14 of spontaneous differentiation are shown as mean with SD from *n* = 3 independent experiments including at least six EBs per experiment and treatment. Where indicated, EpCAM^−/−^ ESCs (#56 and #114) re-expression clones were pre-treated with doxycycline (dox). **C** Expression of α-CAA was quantified by qRT-PCR at day 14 of spontaneous differentiation in EpCAM^−/−^ ESCs upon conditional re-expression of *Eomes*. Shown are mean with SD from *n* = 3 independent experiments performed in triplicates. *p-value < 0.05, ** < 0.01, *** < 0.001, **** < 0.0001. **F**, **G** *Eomes* expression was quantified by qRT-PCR at day seven of spontaneous differentiation in EpCAM^−/−^ ESCs (clones #56 and #114) treated with Mek inhibitor (Meki) (**F**) and in sub-clones re-expressing *Epcam* (**G**). Shown are mean with SD from *n* = 3 independent experiments performed in triplicates. *p-value < 0.05, ** < 0.01, *** < 0.001, n.s. not significant.

In both EpCAM^−/−^ clones, Eomes-EGFP, Foxa2-EGFP, and Gata6-EGFP expression was gradually lost with differentiation (Fig. 5A). In knockout clone #56, Eomes-EGFP leakage in the absence of doxycycline at the time point of EB formation complemented cardiomyocyte formation to 47% of WT. Enhanced expression of Eomes-EGFP upon doxycycline addition greatly restored WT concentration rates in clone #56 at D14 (77% of WT). Weak Eomes-EGFP leakage in clone #114 did not influence contraction rates in the absence of doxycycline but was restorable to 62% of WT after doxycycline-mediated induction (Fig. 5B). α-CAA expression levels were used as a molecular surrogate for the observed functional complementation and confirmed contraction rates observed after Eomes-EGFP re-expression in EpCAM^−/−^ clones (Fig. 5C). Single Foxa2-EGFP re-expression complemented cardiomyocyte formation only in EpCAM^−/−^ clone #56 but not #114, and single Gata6 re-expression had no measurable effects on the contraction of any EpCAM^−/−^ clone (Fig. 5D, E). Consequently, selective re-expression of *Eomes* was observed after MEK inhibition and EpCAM re-expression (Fig. 5F, G). Thus, *Epcam* and *Eomes* re-expression in EpCAM^−/−^ clones complemented the deficiency in exit of pluripotency and spontaneous differentiation to cardiomyocytes.

### *Epcam* induces *Eomes* transcription during differentiation

**Fig. 6:**
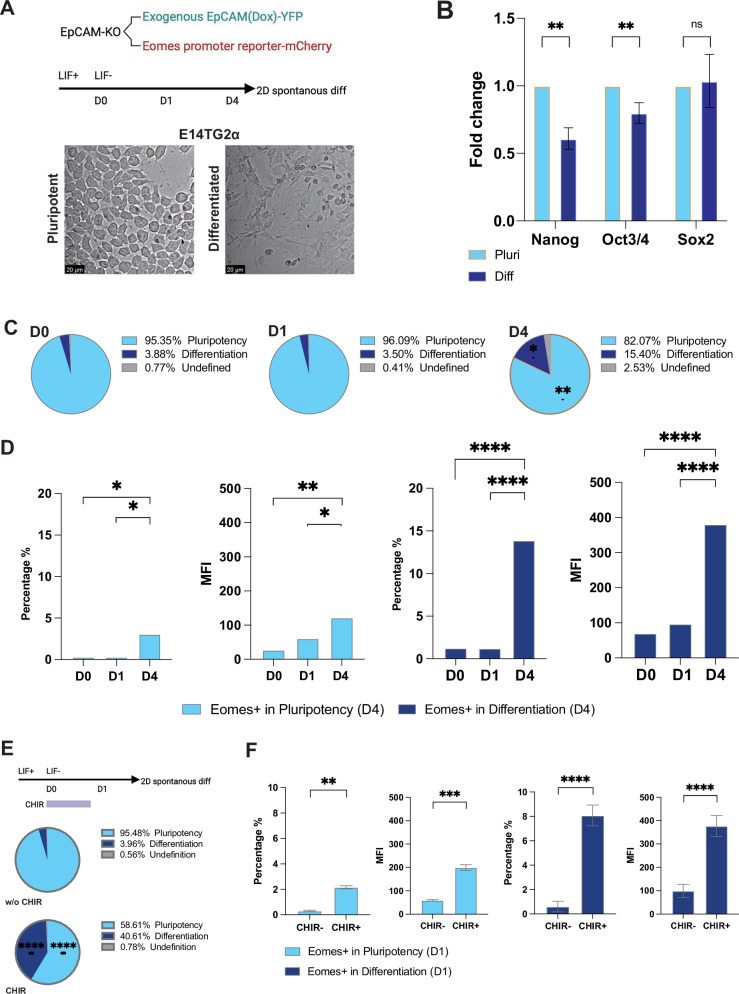
Influence of Wnt signaling on differentiation and Eomes promoter activity. **A** E14TG2α EpCAM−/− ESC clone #114 was stably transfected with a conditional *Epcam* expression vector and an *Eomes* promoter-reporter plasmid expressing mCherry fluorescence protein. ESCs were subjected to 2D spontaneous differentiation via LIF withdrawal. Micrographs of E14TG2α EpCAM−/− ESC morphology) in the presence (pluripotent) and absence (differentiated) of LIF in 2D culture are shown. **B** Pluripotent (Pluri) and differentiated (Diff) mESC were enriched by FACS and the expression of Nanog, Oct3/4, and Sox2 was analyzed by qRT-PCR. Shown are mean and SD of *n* = 3 independent experiments performed in triplicates (** < 0.01). **C** Proportions of pluripotent and differentiated cells are shown as pie charts with mean percentages of cells in pluripotency, differentiation, and in an undefined state from n = 3 independent experiments at D0, 1, and 4 of differentiation in 2D. **D** Quantification of *Eomes* promoter activity as mCherry-positive cells and mean fluorescence intensity in pluripotent and differentiated E14TG2α EpCAM−/− cells at D4 from *n* = 3 independent experiments. Shown are mean with SD. *p-value < 0.05, ** < 0.01, *** < 0.001, **** < 0.0001. **E** E14TG2α EpCAM−/− ESCs harboring a conditional *Epcam* expression vector and an *Eomes* promoter-reporter expressing mCherry were subjected to 2D spontaneous differentiation via LIF withdrawal in the presence or absence of Wnt activator CHIR99021 (CHIR). Differentiation was assessed by flow cytometry using forward scatters of area and height (FSC-A vs. FSC-H). and proportions of cells are given as pie charts (*n* = 3 independent experiments. **F** Quantification of *Eomes* promoter activity as percentage of mCherry-positive cells and mean fluorescence intensity in control- and CHIR99021-treated cells (CHIR- and CHIR + ) from *n* = 3 independent experiments. Shown are mean with SD. One-way ANOVA with post-hoc multiple comparisons: *p*-value ** < 0.01, *** < 0.001, **** < 0.0001.

*Eomes* is a T-box TF orchestrating exit from pluripotency and lineage specification towards mesoderm and definitive endoderm. Thereby, Eomes represents a central regulator of early embryonic development that links early developmental signaling to germ layer specification [35]. We aimed at understanding underlying regulatory mechanisms of *Eomes* as a key mediator of *Epcam*-dependent effects on differentiation. We focused on Wnt signaling that was affected by *Epcam* knockout and sharply increased at the transition from early to late primitive streak cells in scRNAseq data. To interrogate regulatory effects of Wnt signaling and EpCAM on *Eomes* transcription, EpCAM^−/−^ clone #114 bearing a conditional *Epcam-EGFP* expression plasmid was stably transfected with a reporter plasmid consisting of the *Eomes* promoter driving the expression of mCherry as detectable marker. The resulting cell line was subjected to 2D spontaneous differentiation via LIF withdrawal and was monitored by flow cytometry using forward scatters of area and height (FSC-A vs. FSC-H). Differentiation resulted in characteristic morphological changes including elongated, spindle-shaped cells and in the reduction of pluripotency markers Nanog and Oct3/4 (Fig. 6A, B). Over time, proportions of differentiated cells increased along with mCherry-positive cells reflecting *Eomes* promoter activity (Fig. 6C, D). mCherry-positive cells represented 16.99% of all cells at day 4 and 85% were allocated to differentiated cells (Fig. 6D). The influence of Wnt signaling on differentiation and *Eomes* transcription was tested using Wnt activator CHIR99021. Wnt activation promoted mESC differentiation, affecting 40.61% of cells versus 3.96% in the absence of CHIR99021 (Fig. 6E). *Eomes* promoter activity was induced in parallel to cell differentiation with 10.24% of total positive cells and 78.9% of expression in differentiated cells (Fig. 6F, Supplementary Fig. 12).

**Fig. 7:**
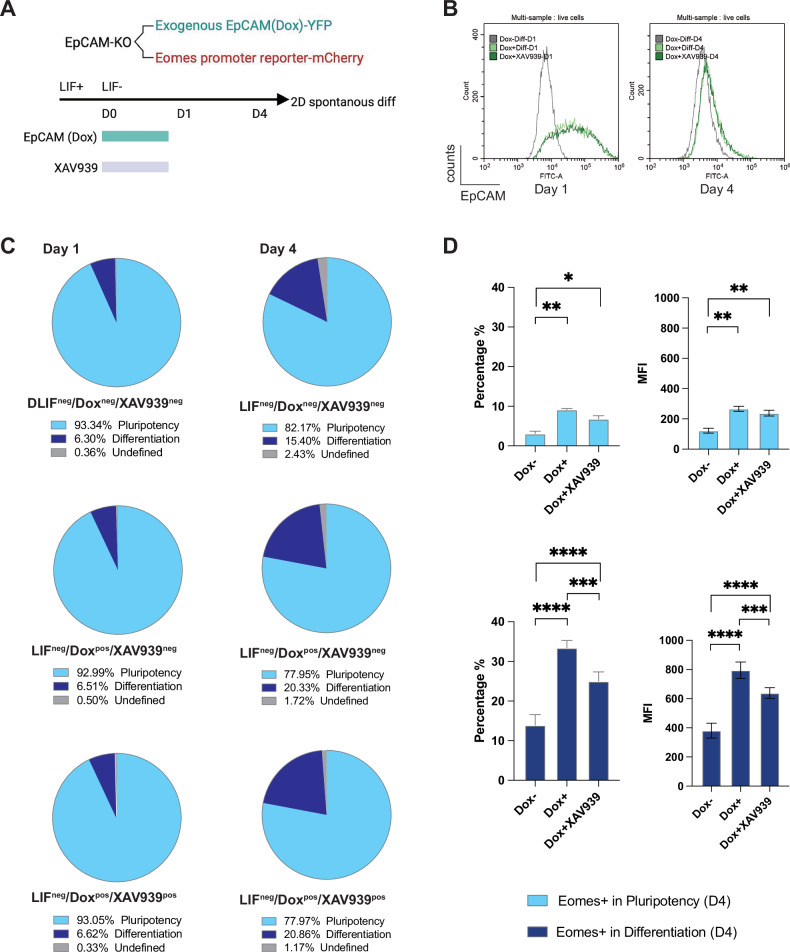
*Epcam* influence on *Eomes* expression. **A** E14TG2α *Epcam*^−/−^ ESCs (clone #114) harboring a conditional EpCAM-EGFP expression vector and an *Eomes* promoter-reporter expressing mCherry were subjected to 2D spontaneous differentiation via LIF withdrawal in the presence or absence of doxycycline for 24 h to induce EpCAM-EGFP expression and of Wnt inhibitor XAV939. **B** EpCAM-EGFP expression was monitored by flow cytometry at D1 and 4. Shown are representative histograms from *n* = 3 independent experiments with cells without doxycycline (gray line), with doxycycline for 24 h (light green), and with doxycycline and XAV939 (dark green). **C** E14TG2α *Epcam*^−/−^ ESCs differentiation was assessed by flow cytometry using forward scatters of area and height (FSC-A vs. FSC-H) at D1 and 4 from *n* = 3 independent experiments. Proportions of pluripotent and differentiated cells are given as pie charts. **D** mCherry expression was assessed by flow cytometry in pluripotent and differentiated ESCs. Quantification of *Eomes* promoter activity as mCherry-positive cells (left) and mean fluorescence intensity (right) in pluripotent and differentiated E14TG2α EpCAM^−/−^ ESCs (clone #114) from *n* = 3 independent experiments. Dox-: control without doxycycline, Dox + : doxycycline treatment (24 h), Dox+ XAV939: doxycycline and XAV939 treatment (24 h). Shown are mean with SD. One-way ANOVA with post-hoc multiple comparisons: *p-value < 0.05, ** < 0.01, *** < 0.001, **** < 0.0001.

Next, EpCAM-EGFP expression was induced via doxycycline in EpCAM^−/−^ clone #114 equipped with an *Eomes*-mCherry reporter without LIF for 24 h in the absence and presence of Wnt inhibitor XAV939 (Fig. 7A). Following a measurable induction of EpCAM-EGFP expression at day 1, EpCAM-EGFP was lost over time and was not influenced by XAV939 (Fig. 7B). No significant differences in pluripotent versus differentiated cell percentages were observed at day 1 based on treatment (average range of 6.30-6.62% of differentiated cells) (Fig. 7C) and the *Eomes* promoter was inactive (Supplementary Fig. 13A, B). Spontaneous differentiation of EpCAM^−/−^ clone #114 in any cell lineage was increased at D4 to an average 15.4% and was further supported by EpCAM-EGFP re-expression (20.33%). XAV939 had no additional effect, yielding proportions of differentiated cells comparable to doxycycline-treated samples (20.86%) (Fig. 7C). Specifically monitoring *Eomes* promoter activity revealed a significant up-regulation upon re-expression of EpCAM-EGFP in pluripotent and, more pronouncedly, in differentiated cells (33.4% positive cells). EpCAM-induced upregulation of the *Eomes* promoter was partially counteracted upon Wnt inhibition and was reduced to 25% (Fig. 7D, Supplementary Fig. 13C, D). We conclude that re-expression of EpCAM in ESC re-activated *Eomes* transcription, partially via Wnt-dependent pathways.

## Discussion

During embryonic development, mesodermal lineages efficiently suppress *Epcam* expression at the onset of gastrulation in the primitive streak, while endodermal lineages express EpCAM [14, 15, 36]. Mechanisms involved in this selective expression were partly uncovered in hESCs, where EpCAM^−^/CD56^+^ early mesodermal progenitors are the foundation of all subsequent mesodermal lineages [36]. Oppositely, B-cell receptor-associated protein 31 BAP31 colocalized with and up-held EpCAM expression along with a state of proliferation and repression of differentiation of hESCs [37]. In concordance with our present findings, human iPSC down-regulate EpCAM expression after primitive streak induction at the bifurcation between endo- and mesodermal lineages, resulting in EpCAM-positive endoderm and EpCAM-negative mesoderm [14]. Mechanistically, *Epcam* gene repression was attributed to histone modifications, primarily trimethylation of lysine 27 in the promoter region by chromatin remodelers SUZ12 and JMJD3 [19]. The reduction of EpCAM protein during murine guided mesodermal ESC differentiation and in human carcinoma cells undergoing EMT is mediated by enhanced endocytosis and reduced membrane recycling via binding to differential Rab proteins [25]. Hence, regulatory mechanisms are implemented at various levels governing EpCAM expression and in multiple species including human, murine, porcine, xenopus, and zebrafish cells [9, 10, 15, 21, 26, 38–43].

**Fig. 8:**
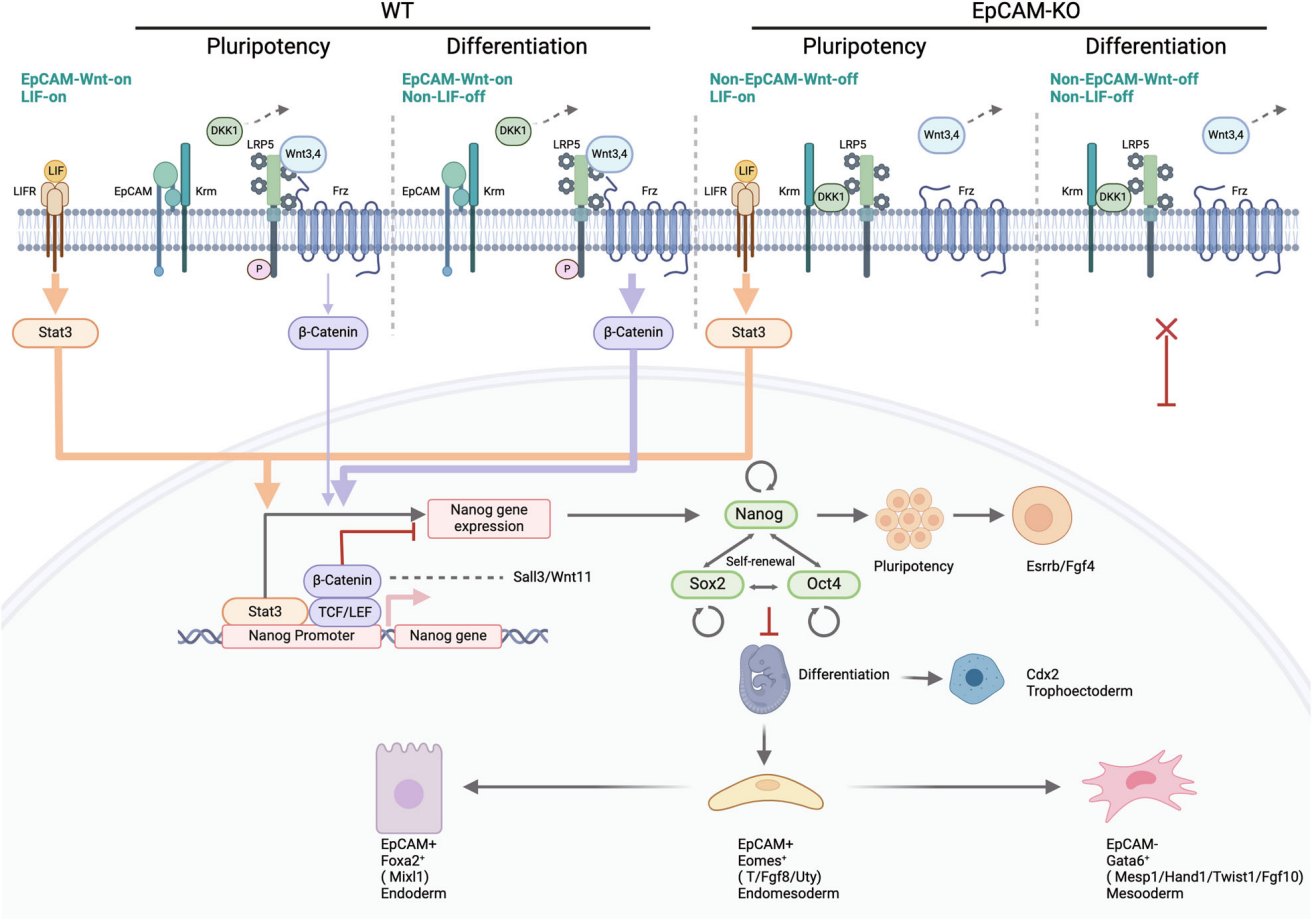
Schematic representation of molecular interactions of EpCAM and Wnt signaling Components in mESC under pluripotency and differentiation. Shown is a schematic representation of the signaling interplay of EpCAM and Wnt signaling in pluripotency and differentiation of murine embryonic stem cells. Interactions include ligand-receptor pairs invovlved in downstream cascades leading to Stat, β-Catenin, Nanog, Sox2, Oct4 and the regulation of a pluripotent state or, alternatively, exit from pluripotency and induction of lineage specificity. Interactions are visualized in the presence and absence of EpCAM (wildtype: WT; CRISPR-Cas9-medicated Epcam knockout: EpCAM-KO).

Downstream effects of EpCAM in differentiation are less well understood. Early studies in mESCs suggested a linkage of EpCAM to the expression of c-Myc, Sox2, Oct-4, and Stat3, a reduction of proliferation and the induction of endo- and mesodermal markers upon silencing of EpCAM [9, 10]. The binding of the intracellular signaling domain of EpCAM (EpICD) to regulatory DNA elements of c-Myc, Oct-4, Nanog, Sox2, and Klf4 is enhanced under pluripotency and is gradually lost during differentiation of human ESCs [19]. In the present study, effects of a genetic knockout of EpCAM under pluripotency conditions were minor and did not support a major function in maintenance of pluripotency, except for a down-regulation of *c-Myc*. Activation of LIF receptor under pluripotency conditions induces Stat3, which plays a central role in maintenance of pluripotency by activating *Nanog* expression and by transcriptional cooperation with Nanog, Sox2, and Oct4 [44–46]. It is conceivable that such regulatory circuit overpowers EpCAM-mediated effects under pluripotency conditions and therefore masks effects of the genetic loss of *Epcam* (Fig. 8).

Augmented temporal resolution of transcriptomic changes in mESC differentiation revealed three major phases. Early and late pluripotency occur within the initial 24-48 h of differentiation in EBs, respectively, and are followed by molecular features of primitive streak formation (72 h) and subsequent cell specialization (96 h and beyond) [5]. Our time-resolved bulk and scRNA sequencing analyses demonstrated a role of *Epcam* in exit from pluripotency and progression of cell differentiation via linkage to several key transcription factors, receptors, and ligands. Exit from pluripotency was accompanied by an initial increase in *Epcam* expression, followed by a sharp loss, reminiscent of human iPSCs [14]. Primitive streak formation at 72 h is characterized by an upregulation of *Eomes*, *Brachyury* (*T*), *Mixl1*, and *Evx1*, coinciding with peaking co-expression of *Epcam*. Upon LIF withdrawal, effects of increased EpCAM on Wnt signaling may become relevant in the repression of key stemness genes such as *Nanog* and the induction of differentiation-regulating transcription factors (Fig. 8). In Zebrafish EpCAM serves as a scavenger for Kremen1-DKK2 to de-repress Wnt signaling in liver progenitors [47]. thus supporting its role in the hepatic endodermal lineage [14] via Wnt regulation. EpCAM interaction with Kremen1 and DKKs may further impact on ESC survival via Wnt-independent mechanisms, since Kremen1 reportedly acts as apoptosis-inducing receptor [48].

Expression of key primitive streak formation and gastrulation transcription factors *Brachyury*, *Eomes*, *Mixl1*, and *Twist1*, as well as of central regulators of cell fate (*Nanog*, *Dkk1*, *Foxa2*, *Gata6*, *Hand1*, *Mesp1*, and *Sall3*) was significantly reduced in EpCAM^−/−^ ESC. Thus, loss of EpCAM was prominently linked to a concurrent lack of induction of numerous transcriptional regulators of primitive streak formation such as *Eomes*, *Brachyury*, *Mixl1* [49–53]. endo- and mesoderm (*Foxa2*, *Gata6*) [54, 55]. and cardiomyocytic differentiation (*Mesp1*) [56, 57]. scRNAseq analysis of wildtype mESC corroborated such a role of *Epcam* in the regulation of *Eomes* at the transition of early to late primitive streak via Wnt activation. Pseudotime analysis of differentiating mESCs validated the postulated *Epcam*/Wnt/*Eomes* axis in cardiomyocyte formation at single cell-level and its implication in gastrulation. The reduction of markers for cardiomyocyte formation such as *α-Caa* in *Epcam*^−/−^ cells most likely represents a downstream consequence of the impairment of this *Epcam*/Wnt/*Eomes* axis. *Eomes* is a pivotal regulator of early mesoderm and cardiac progenitor formation and operates upstream of the cardiogenic cascade comprising *Eomes*, *Mesp1*, *Mixl1*, *Nkx2-5*, and sarcomeric genes including *α-Ca*a [35, 53, 58]. Consistent with this framework, loss of *Epcam* lowers *Eomes*, which attenuates the activation of the *Mesp1*/*Mixl1* module and the subsequent *Nkx2-5*-dependent program, ultimately resulting in decreased expression of cardiac structural genes such as *α-Caa*.

The lack of TF induction in *Epcam*^−/−^ clones was paralleled by increased expression of cycline-dependent kinase inhibitor *Cdkn2a* that encodes cell cycle inhibitors p16(INK4A) and p14(ARF). p16-dependent senescence was demonstrated to limit cellular plasticity during the generation of induced pluripotent stem cells and totipotent cells [59, 60]. Hence, *Cdkn2a* upregulation may further reduce exit of pluripotency and limit differentiation observed in EpCAM^−/−^ mESC.

Conditional expression of EpCAM complemented defects in cardiomyocyte formation, provided it mimicked its natural expression dynamic of a strong initial expression followed by sharp loss. Re-expression of EpCAM restored *Eomes* expression and the contraction capacity of EBs close to wildtype levels. Co-expression of *Epcam* and *Eomes* in early stages of murine embryogenesis and at early stages of spontaneous differentiation in EBs corroborated an interdependency of both genes at single-cell level and in an inferred pseudotime stream of differentiation. Wnt activators and inhibitors provided further evidence for a role of Wnt signaling in the induction of differentiation, EpCAM-mediated *Eomes* transactivation, and cardiomyocyte formation. These findings are in line with a reported function of Wnt-mediated *Eomes* regulation during cardiac induction in hESCs, which follows a dynamic expression during ESC differentiation comparable to *Epcam*. Wnt-driven *Eomes* expression is initially required to suppress the mesoderm repressor *Sox2* and initiate mesoderm formation. Thereafter, inhibition of Wnt activity is primordial to allow further lineage specification to cardiomyocytes through the restriction of cardiac repressors *Msx1* and *Cdx2* [61]. Consequently, *Epcam*^−/−^ ESC showed enhanced *Cdx2* expression at later time points of spontaneous differentiation (D7), supporting the model of an *Epcam*/*Wnt*/*Eomes*-axis promoting mESC differentiation to cardiomyocytes. Accordingly, the knockout of *Epcam* recapitulates an *Eomes* deletion. ESCs harboring an *Eomes* deletion retain their characteristic morphology, express pluripotency markers Nanog, Oct4/4, and Sox2, but fail to form mesodermal progeny and are incapable of generating cardiac mesoderm and definitive endoderm [53, 58]. Hence, lack of *Epcam* and *Eomes*, both, negatively impact on the ability of ESCs to exit pluripotency.

Effects of EpCAM on differentiation via additional signaling pathways is likely, given the broad range of reported activities of EpCAM. Particularly, a regulation of the MAPK/Erk1/2 pathway by EpCAM has been demonstrated in tumors and in stem cells [18, 22, 23, 62, 63]. It is therefore worth mentioning that, despite clear reports of Wnt activation via MEK inhibitors [27, 28], a contribution of Erk1/2 to the complementation of EpCAM^−/−^ ESC cannot be excluded. Such scenario suggests potential relationships between the newly identified *Epcam*/Wnt/*Eomes* axis and additional EpCAM-dependent cellular signals (reviewed in [26]). Association of *Epcam* and fDEGs with elevated EGFR and MAPK signaling at stages of primitive streak formation is in line with the proposed role of EpEX in inducing EGFR signaling in iPSCs [17, 18]. Further EpCAM-dependent regulatory mechanisms comprise signaling pathways involving RIP of EpCAM, interaction with embryonic Ras (ERas), E-cadherin and claudins [15, 17–19, 21, 42, 47]. Both RIP products and ERas-mediated signaling may therefore be instrumental in the *Epcam*/Wnt/*Eomes* axis and correlated PROGENy pathways.

In summary, we provide to our knowledge the first evidence for a function of EpCAM in exit of pluripotency via Wnt-mediated regulation of *Eomes*.

### Limitations of the study

Functional and transcriptomic analyses of the genetic knockout of *Epcam* presented in this study are limited to a 3D model of differentiation. Differences to murine embryogenesis in vivo can therefore not be excluded. This limitation has been partly overcome using public and in-house scRNAseq datasets of murine embryogenesis and differentiation, demonstrating correlations of *Epcam* expression with relevant TFs at the single-cell level. Future work should therefore aim at a validation of the presented Epcam/Wnt/Eomes axis in vivo.

## Materials and Methods

### Biological and technical replicates, statistical analysis

Throughout the manuscript, biological replicate is referred to as a fully independent experiment performed with newly generated materials, whereas a technical replicate is a repeated measurement with identical materials. Samples sizes are indicated for each experiment in the cognate figure legends and comprise a minimum of *n* = 3 biological replicates. Bulk and scRNAseq experiments were generated in quadruplicates. Sample exclusion was performed for RNA sequencing samples based on obvious divergence in transcriptomic profiles (*n* = 1000 most differential genes) and was applied for one sample of bulk seq data of wild-type cells at D3. Statistical analysis of comparisons of two samples with similar variances within samples was performed with an unparied student t-test where a *p*-value ≤ 0.05 was considered significant. Statistical analysis of comparisons of multiple samples was performed with a one-way ANOVA and *post-hoc* multiple testing with Tuckey or Bonferroni corrections. *P*-values ≤ 0.05 were considered significant.

### Cell lines, cell culture and stable cell transfection

Mouse embryonic stem cells (ESC) E14TG2${\rm{\alpha }}$ were cultured in Mouse ES Cell Basal Medium (ATCC, Manassas, Virginia, USA), 2-mercaptoethanol (Gibco™; 10 nM; Paisley, Scotland) and leukemia inhibitory factor (ESGRO®LIF; 1000 U/mL; Becton Dickinson, Heidelberg, Germany) supplemented with 10% FBS (Bio&SELL, Feucht, Germany) on 0.1% gelatin (InSCREENeX, Braunschweig, Germany) coated T25 flasks. E14TG2${\rm{\alpha }}$ EpCAM knockout #56 and #114 are independent single-cell clones generated using guide-RNAs targeting exon 2 and exon 4, respectively [15]. Clone #56 has a 13 bp deletion leading to a truncated putative protein of 35 aa. Clone #114 carries a 13 bp deletion putatively generating a 134 aa product. Both putative protein products miss the transmembrane domain and would therefore not be anchored in the plasma membrane to deploy their function. EpCAM-specific antibodies target the very N-terminus and failed to detect any protein product. EpCAM knockout clones were transfected with mEpCAM-EGFP, mEomes-EGFP, mFoxa2-EGFP, mGata6-EGFP in a PiggyBac expression backbone driven by a doxycycline-responsive promoter with a blasticidin selectable marker. This vector was derived from pFH2.94_PB-TRE3GS-Gag-MCP-T2A-eGFP-PGK-Tet3G-Blast (Addgene #205540) by replacing the Gag-MCP-T2A-eGFP cassette with each gene of interest. The vector ensures stable genomic integration and long-term maintenance of inducible transgene expression [64]. Consistent results from Western blot, flow cytometry, and immunofluorescence analyses performed at multiple time points confirmed stable transgene expression without variation in protein size, localization, or induction efficiency. Cells were selected and cultivated with 12.5 ng/μl blasticidin (Invivogen, San Diego, USA). Cond.EpCAM-#114 cells were transfected in a 6-well plate with *mEomes* promoter reporter-mCherry in the pEZX-PM02 backbone containing a puromycin selectable marker and then continuously selected with 2.5 ug/ml puromycin (Invivogen, San Diego, USA) and 12.5 ng/ul blasticidin.

### Spontaneous and directed 2D differentiation

ESC (100,000 cells/well) were seeded in 6-well plates in medium w/o LIF, 37 °C, 5% CO_2_. For Wnt activation and inhibition, 30 μM CHIR 99021 (Sigma, St.Louis, MO, USA) and 10 μM XAV-939 (Sigma, St.Louis, MO, USA) were supplemented for 24 h, respectively. Cells were washed with PBS and continuously differentiated in medium w/o LIF. At time points day 1, 4, and 7, cells were harvested for further analysis.

### Embryoid bodies (EBs) formation and contraction

To generate EBs, 500 cells in 20 µl of LIF-free medium were plated on the lid of a 150 mm tissue culture dish and cultured as hanging drops by inverting the lid. After three days, EBs were transferred to ultra-low attachment plates (Nunc, Wiesbaden, Germany) for four days before transfer to standard 96-well plates for further differentiation up to 14 days. During this period, 100 ul of fresh culture medium was replaced every two days. Contraction of EBs was analyzed after 10–14 days by counting under a microscope in 96-well plates. Contraction rate represents the percentage of contracting EBs standardized to the number of EBs formed.

### Treatment with Wnt11 and Wnt-regulating compounds, cryo-preservation and sectioning

EpCAM-KO EBs were allowed to differentiate spontaneously for 14 days and were treated with chemicals and proteins for the initial six days, including 1 µM MEK inhibitor AZD6244 (Selleckchem, Munich, Germany), 25 nM GSK3β inhibitor CHIR99021, 50 nM DKK1 inhibitor WAY (Sigma-Aldrich, Steinheim, Germany), and 0.75 ug/ml Wnt11 (Bio-techne, Minnesota, USA) before assessing contraction rates at D14. EBs from D3, D5, D7, D14 were embedded in tissue-tek (Sakura Finetek, Germany), snap-frozen in liquid nitrogen, cut into 4 μm thick sections, and stored at −20 °C.

### Immunohistochemistry (IHC) and Immunofluorescence (IF)

Immunohistochemistry and immunofluorescence staining of EpCAM (BD552370, 1:500, Abcam, Waltham, USA), α-CAA (AC1-2042, 1:200, Sigma, Germany), Foxa2 (#8186, 1:400, Cell Signaling, Leiden, The Netherlands), Eomes (ab216870, 1:200, Abcam, Waltham, USA), Gata6 (55435-1-AP, 1:500, Proteintech®, Planneg-Martinsried, Germany) was performed using avidin-biotin-peroxidase complex method (Vectastain, Vector laboratories, Burlingma, CA, USA) and Alexa Fluor-488- and Alexa Fluor-647-conjugated secondary antibodies (Invitrogen, Thermo Fisher, Darmstadt, Germany) respectively. Confocal microscopy imaging was conducted with a TCS-SP8 scanning system and a DM-IRB inverted microscope (Leica, Nussloch, Germany).

### Flow cytometry (FACS)

Single cell suspension of EBs was generated by treatment with 1 mL Accutase (Capricorn-scientific, Hesse, Germany) (1 min, RT), neutralization with culture medium, washing with PBS, and filtering with a 40 µm filter (Pluriselect, Leipzig, Germany). Cells were stained with EpCAM-specific antibody (#14-5791-81, Invitrogen, Waltham, USA) for 15 min on ice, washed three times in PBS-3%FBS, and stained with fluorescein isothiocyanate-conjugated rabbit anti-mouse secondary antibody. Measurement of cell surface expression of EpCAM was performed in a CytoFLEX device (Beckman Coulter, Krefeld, Germany).

### Immunoblotting

Immunoblotting of EpCAM, Eomes, Foxa2, Gata6 and β-actin (Santa cruz biotechnology, Dallas, USA) was performed with 10–50 µg whole cell lysate (PBS, 1% triton X-100, Roche complete protease inhibitors) separated in a 10-15% SDS-PAGE, and transferred on PVDF membranes (Millipore, Germany). Primary antibodies were detected with HRP-conjugated secondary antibodies and ECL reagent (Merk Millipore, Darmstadt, Germany). All uncropped immunoblot are available as **Supplementary Figure_immunoblots**.

### Quantitative reverse transcription polymerase chain reaction (RT-qPCR)

Total mRNA was prepared using RNeasy Mini Kit (Qiagen, Hilden, Germany) and reverse transcribed with QuantiTect Reverse Transcription-Kit (Qiagen, Hilden, Germany). cDNA was amplified using SYBR-Green PCR mastermix (Qiagen, Hilden, Germany) and specific primers. Normalization across samples were performed using glucuronidase beta (GUSB) expression. Gene expression levels were calculated according to the equation 2-ΔΔCT, where ΔCT was defined as the difference between CT of interest genes and CT of control genes.

The following primers were used for qRT-PCR (forward/backward):

GUSB: FW CAACCTCTGGTGGCCTTACC; GGGTGTAGTAGTCAGTCACAGAC

Eomes: GGCCTACCAAAACACGGATATC; TTTCTGAAGCCGTGTACATGGA

Foxa2: CCCTACGCCAACATGAACTCG; GTTCTGCCGGTAGAAAGGGA

Gata6: GGTCTCTACAGCAAGATGAATGG; TGGCACAGGACAGTCCAAG

Twist1: CCCACCCCACTTTTTGACGA; CAGTGGCTGATTGGCAAGAC

T: GCTTCAAGGAGCTAACTAACGAG; CGTCACGAAGTCCAGCAAGA

Fgf10: TTTGGTGTCTTCGTTCCCTGT; TAGCTCCGCACATGCCTTC

Mixl1: CTACCCGAGTCCAGGATCCA; ACTCCCCGCCTTGAGGATAA

DKK1: TGAGGGCGGGAACAAGTA; TTCGGCAAGCCAGACAGA

Mesp1: GCTCGGTCCCCGTTTAAGC; ACGATGGGTCCCACGATTCT

α-CAA:CTGGATTCTGGCGATGGTGTA; CGGACAATTTCACGTTCAGCA

Wnt11: GGATATCCGGCCTGTGAAGG; TCCACCACTCTGTCCGTGTA

Fgf8: TGTTGCACTTGCTGGTTCTC; CGGCTGTAGAGCTGGTAGG

Hand1: GTGAGTGCATCCCCAATGTG; GCCAGCACGTCCATCAAGTA

Cdx2: CTGCTGTAGGCGGAATGTATGTCT; AAGGCTTGTTTGGCTCGTTACAC

Esrrb: CGATTCATGAAATGCCTCAA; CCTCCTCGAACTCGGTCA

Fgf4: ACTACCTGCTGGGCCTCAA; ACTCCGAAGATGCTCACCAC

Uty: AGATGAAGACGCTGTTGAAC; CTAATTGCCCACTGAAATGC

Sall3: CCTGATTCTTCCTGGTGGAGT; CTCTGGAAAACGCCACAGAC

Nanog: TCTTCCTGGTCCCCACAGTTT; GCAAGAATAGTTCTCGGGATGAA

### Cloning procedures

PCR amplification of the inserted gene was conducted using Q5® High-Fidelity DNA Polymerase (New England Biolabs, Frankfurt, Germany) in thermal cycler (BioER, Bremgarten, Switzerland). DNA template was purchased from Addgene as bacterial stabs including Tet3G-on-vector (#205540), mEpCAM, mEomes (#200888), mFoxa2(#33014) and mGata6 (#72694) inserts. NEBuilder® HiFi DNA Assembly (New England Biolabs, Frankfurt, Germany) was used to ligate inserted coding sequences and vectors.

The following primers were used for cloning (forward/backward):

mEpCAM: TCGTAAAGCTAGCGGATCCGCCACCATGGCGGGTCCCCAGGCCCT GCCGCCGCCGCCGCCGCCGGCATTAAGCTCTCTGT

mEomes: TCGTAAAGCTAGCGGATCCGCCACCATGCAGTTGGGAGAGCAGCT

GCCGCCGCCGCCGCCGCCGGGACTTGTGTAAAAAGCA

mFoxa2: TCGTAAAGCTAGCGGATCCGCCACCATGCTGGGAGCCGTGAAGAT GCCGCCGCCGCCGCCGCCGGATGAGTTCATAATAGGCC

mGata6: TCGTAAAGCTAGCGGATCCGCCACCATGGCCTTGACTGAG GCCGCCGCCGCCGCCGCCCTGTTCTCGGGGTTGGCG

### RNAseq and differential expression (DE) analysis

RNA was extracted using the RNeasy Mini Kit (Qiagen, Germany) and quantified using Qubit™ RNA BR Assay Kit (#Q10210) with a Qubit Fluorometer (Thermo Fisher Scientific, MA, USA). RNA sequencing libraries were prepared with 100 ng total RNA input using the QuantSeq 3′ mRNA-Seq Library Prep Kit FWD for Illumina (#SKU:015.96; Lexogen, Austria). For library amplification, PCR cycles were determined with the PCR Add-on Kit for Illumina (#SKU:020.96, Lexogen) and individual libraries were amplified with 18 PCR cycles. Quality and quantity of the libraries were evaluated using Quanti-iT PicoGreen dsDNA Assay Kit (P7589, Thermo Fisher) and Bioanalyzer High Sensitivity DNA Analysis Kit (#5067-4626, Agilent Technologies). Libraries were sequenced with 150 bp paired-end mode on a HiSeq400 sequencer (Illumina, Germany). 3´-Bulk RNAseq data are available as GSE 293231 at GEO (https://www.ncbi.nlm.nih.gov/geo/).

Differential expression (DE) analysis of bulk RNA-seq data was performed using DESeq2 [65]. Wild-type (WT) cells served as controls and were compared to *Epcam* knockout lines (#114 and #56) at D0, D3, D7, and D10. DE genes (DEGs) were identified based on thresholds of |log2 fold change | > 1.0 and FDR ≤ 0.05. Volcano plots represents the up and down-regulated DEGs of differentiation stages between *Epcam* knockouts vs. WT. Gene set enrichment analysis (GSEA) was conducted on genes ranked by fold change and visualized with clusterProfiler package (Bioconductor) in R. Enriched gene sets were cross-referenced with canonical pathways (CP) from the Molecular Signatures Database (MSigDB; https://www.gsea-msigdb.org/gsea/msigdb), focusing on pluripotency, mesoendoderm differentiation, mesoderm and ectoderm differentiation, heart development, cardiac contraction, and Wnt signaling pathways. Signature genes were identified by intersecting the gene list from DEGs, GSEA, and MSigDB hallmark pathways of differentiation stages. Venn diagrams were used to visualize common genes across comparisons.

### Public single cell RNA-sequencing dataset analysis

Single-cell RNA-sequencing datasets are available at GSE100597 (GEO) and https://github.com/MarioniLab/EmbryoTimecourse2018, respectively. GSE100597 contains scRNA-seq data from mouse embryos at gestation days E3.5 (*n* = 10 embryos, *n* = 99 cells), E4.5 (*n* = 5 embryos, *n* = 105 cells), E5.5 (*n* = 9 embryos, *n* = 267 cells), and E6.5 (*n* = 11 embryos, *n* = 250 cells) [29]. The dataset from Pijuan-Sala et al. contains scRNA-seq data 116,312 cells from gestation day E6.5-E8.5 [30]. Data was visualized with uniform manifold approximation and projection (UMAP) in R (https://www.r-project.org/, version R 4.3.0).

Publicly available preprocessed single-cell RNA sequencing data were integrated to explore lineage and regulatory processes across species and developmental stages. Single-cell data from C57Bl/6Babr mouse embryos (GSE100597) at stages E3.5, E4.5, E5.5, and E6.5 were processed with Seurat package in R for data normalization, log2 transformation, and scaling. Embryonic developmental cell lineages score was calculated using the *AddModuleScore* function in Seurat [66]. Additionally, single cell transcriptional profiles of 116,312 cells from C57BL/6 mice embryos (E6.5 to E9.5) [30].were analyzed using the Scanpy package in Python [67]. Preprocessed human embryogenesis (GSE157329) single-cell data from embryos at Carnegie stages (CS) 12–16 (4–6 weeks) and across 18 developmental systems based on over 180,000 transcriptomes [68]. were analyzed using Scanpy. Single cell datasets are visualized for embryonic stages and identified cell lineages. Expression patterns of signature genes were mapped across developmental stages and cell lineages, followed by analysis of co-expressed signature genes.

### Single cell RNA capture, library prep, and sequencing

Single cell RNA sequencing was performed with the BD Rhapsody HT single cell analysis system (BD Biosciences, Heidelberg, Germany) using adapted protocols. Wildtype murine embryonic stem cells E14TG2α were harvested in four individual consecutive passages to form embryoid bodies for spontaneous differentiation in the absence of LIF for three, five, and seven days (D3, D5, D7) generating a total of *n* = 12 samples. EBs of each biological replicate were collected in separate reservoir tanks, transferred to 50 mL Falcon tubes, and sedimented via gravity before carefully removing supernatant. EBs were resuspended in 2 mL of TrypLE solution and incubated with constant mixing at 37 °C for 1–2 min to generate single cell suspensions. Cells from each replicate and time point were separately centrifuged 5 min at 400 g, washed once with PBS with 1% FBS (FACS buffer). From here on, all steps were conducted on ice. Custom BD^TM^ Single Cell Multiplexing Kit (#626545; anti-mouse MHC-calls I antibody, direct labelling) was used for sample tagging and pooled processing according to the manufacturer´s protocol. Labelled cells from four replicates (each 2,5 × 10^5^ cells) were pooled for each individual time point to three samples (D3, D5, D7) and washed twice in 2 mL of FACS buffer, centrifuged at 400 g for 5 min, and finally resuspended in 620 μL cold sample buffer. Staining cells with viability markers was achieved by adding 3.1 μL of 2 mM Calcein AM and 3.1 μL of 0.3 mM DRAQ7™ to the cell suspension in cold sample buffer (37 °C in the dark, 5 min). 10 µL cells were counted using BD Rhapsody scanner 10 μL in an INCYTO™ disposable hemocytometer with the remaining cells kept on ice and protected from light. Then, BD Rhapsody 8 lane cartridge were primed, loaded with labelled cells and, thereafter, with BD Rhapsody™ Enhanced Cell Capture Beads. After cell lysis and release of polyadenylated mRNAs, BD Rhapsody™ Enhanced Cell Capture Beads were retrieved, washed, and processed for library preparation. Reverse transcription was performed with the BD Rhapsody^TM^ cDNA kit (#633773) and library preparation with the BD Rhapsody^TM^ Whole Transcriptome Analysis Amplification kit (#633801), generating Illumina-compatible libraries. cDNA library preparation and sequencing as paired-end reads were performed by the group of Dr. Inti Alberto De La Rosa Velásquez of the Core Facility Genomics (Helmholtz Center Munich, Germany) on an Illumina NovaSeq X Plus sequencer in a 10B200 flow cell. The scRNAseq dataset is available under GSE318465 at the Gene Expression Omnibus (https://www.ncbi.nlm.nih.gov/geo/).

### In-house scRNAseq data analysis

In-house scRNAseq data were processed using the Seurat package in R. Low-quality cells and doublets were removed through standard quality control process. Subsequently, 2000 highly variable genes were identified for downstream analysis. Harmony analysis was employed to remove batch effects. Cell clustering analysis was performed using *FindClusters* and *FindNeighbors* functions. Pearson correlation analysis was conducted to estimate the association between selected genes. PROGENy [69]. was used to assess pathway activities based on perturbation-derived gene signatures at the single-cell level and subsequently compared across cell populations or between different genes. Differential pathway activities were analyzed to depict signaling dynamics during cellular state transitions and the associations of individual genes with signaling pathways.

Diffusion pseudotime (DPT) analysis was performed to infer cellular trajectories based on diffusion maps constructed from the normalized single-cell expression data [32]. Epiblast cell population were specified as the root for pseudotime calculation, and cells were ordered along the inferred developmental trajectory. scTour was performed to construct cellular trajectories by learning latent temporal representations from the normalized single-cell expression data [33].

## Supplementary information

Original Data

Supplementary Figures

Supplementary Table 1

41419_2026_8734_MOESM4_ESM

## Data Availability

All codes and R-packages used in the study are publicly available and have been disclosed in Methods or are available from the corresponding authors on reasonable request. RNA sequencing data have been uploaded at the Gene Expression Omnibus (GEO), GSE 293121, GSE318465. Additional materials are available upon request from the authors.

## Code availability

All codes used in the present study are mentioned in the cognate section of data analysis and are public domain.

## References

[1] Evans M. Discovering pluripotency: 30 years of mouse embryonic stem cells. Nat Rev Mol Cell Biol. 2011;12:680–6. 21941277 10.1038/nrm3190

[2] Evans MJ, Kaufman MH. Establishment in culture of pluripotential cells from mouse embryos. Nature. 1981;292:154–6. 7242681 10.1038/292154a0

[3] Martin GR. Isolation of a pluripotent cell line from early mouse embryos cultured in medium conditioned by teratocarcinoma stem cells. Proc Natl Acad Sci USA. 1981;78:7634–8. 6950406 10.1073/pnas.78.12.7634 PMC349323

[4] Desbaillets I, Ziegler U, Groscurth P, Gassmann M. Embryoid bodies: an in vitro model of mouse embryogenesis. Exp Physiol. 2000;85:645–51. 11187960

[5] Gloss BS, Signal B, Cheetham SW, Gruhl F, Kaczorowski DC, Perkins AC, et al. High resolution temporal transcriptomics of mouse embryoid body development reveals complex expression dynamics of coding and noncoding loci. Sci Rep. 2017;7:6731. 28751729 10.1038/s41598-017-06110-5 PMC5532269

[6] Boheler KR, Czyz J, Tweedie D, Yang HT, Anisimov SV, Wobus AM. Differentiation of pluripotent embryonic stem cells into cardiomyocytes. Circulation Res. 2002;91:189–201. 12169644 10.1161/01.res.0000027865.61704.32

[7] Doevendans PA, Kubalak SW, An RH, Becker DK, Chien KR, Kass RS. Differentiation of cardiomyocytes in floating embryoid bodies is comparable to fetal cardiomyocytes. J Mol Cell Cardiol. 2000;32:839–51. 10775488 10.1006/jmcc.2000.1128

[8] Went PT, Lugli A, Meier S, Bundi M, Mirlacher M, Sauter G, et al. Frequent EpCam protein expression in human carcinomas. Hum Pathol. 2004;35:122–8. 14745734 10.1016/j.humpath.2003.08.026

[9] Ng VY, Ang SN, Chan JX, Choo AB. Characterization of Epithelial Cell Adhesion Molecule as a Surface Marker on Undifferentiated Human Embryonic Stem Cells. Stem Cells. 2010;29–5. 10.1002/stem.221 19785009

[10] Gonzalez B, Denzel S, Mack B, Conrad M, Gires O. EpCAM Is Involved in Maintenance of the Murine Embryonic Stem Cell Phenotype. Stem Cells. 2009;27:1782–91. 19544432 10.1002/stem.97

[11] Gires O, Klein CA, Baeuerle PA. On the abundance of EpCAM on cancer stem cells. Nat Rev Cancer. 2009;9:143. 19132011 10.1038/nrc2499-c1

[12] Dolle L, Theise ND, Schmelzer E, Boulter L, Gires O, van Grunsven LA. EpCAM and the biology of hepatic stem/progenitor cells. Am J Physiol Gastrointest Liver Physiol. 2015;308:G233–50. 25477371 10.1152/ajpgi.00069.2014 PMC4329473

[13] Conigliaro A, Amicone L, Costa V, De Santis Puzzonia M, Mancone C, Sacchetti B, et al. Evidence for a common progenitor of epithelial and mesenchymal components of the liver. Cell Death Differ. 2013;20:1116–23. 23686136 10.1038/cdd.2013.49 PMC3705603

[14] Galdos FX, Lee C, Lee S, Paige S, Goodyer W, Xu S, et al. Combined lineage tracing and scRNA-seq reveals unexpected first heart field predominance of human iPSC differentiation. Elife. 2023;7:12:e80075. 10.7554/eLife.80075 PMC10348749 37284748

[15] Sarrach S, Huang Y, Niedermeyer S, Hachmeister M, Fischer L, Gille S, et al. Spatiotemporal patterning of EpCAM is important for murine embryonic endo- and mesodermal differentiation. Sci Rep. 2018;8:1801. 29379062 10.1038/s41598-018-20131-8 PMC5789065

[16] Chen HF, Chuang CY, Lee WC, Huang HP, Wu HC, Ho HN, et al. Surface Marker Epithelial Cell Adhesion Molecule and E-cadherin Facilitate the Identification and Selection of Induced Pluripotent Stem Cells. Stem Cell Rev. 2011;722−35. 10.1007/s12015-011-9233-y 21305366

[17] Kuan II, Lee CC, Chen CH, Lu J, Kuo YS, Wu HC. The extracellular domain of epithelial cell adhesion molecule (EpCAM) enhances multipotency of mesenchymal stem cells through EGFR-LIN28-LET7 signaling. J Biol Chem. 2019;294:7769–86. 30926604 10.1074/jbc.RA119.007386 PMC6514611

[18] Kuan II, Liang KH, Wang YP, Kuo TW, Meir YJ, Wu SC, et al. EpEX/EpCAM and Oct4 or Klf4 alone are sufficient to generate induced pluripotent stem cells through STAT3 and HIF2alpha. Sci Rep. 2017;7:41852. 28157205 10.1038/srep41852 PMC5291097

[19] Lu TY, Lu RM, Liao MY, Yu J, Chung CH, Kao CF, et al. Epithelial cell adhesion molecule regulation is associated with the maintenance of the undifferentiated phenotype of human embryonic stem cells. J Biol Chem. 2010;285:8719–32. 20064925 10.1074/jbc.M109.077081 PMC2838295

[20] Maetzel D, Denzel S, Mack B, Canis M, Went P, Benk M, et al. Nuclear signalling by tumour-associated antigen EpCAM. Nat Cell Biol. 2009;11:162–71. 19136966 10.1038/ncb1824

[21] Hachmeister M, Bobowski KD, Hogl S, Dislich B, Fukumori A, Eggert C, et al. Regulated intramembrane proteolysis and degradation of murine epithelial cell adhesion molecule mEpCAM. PLoS One. 2013;8:e71836. 24009667 10.1371/journal.pone.0071836 PMC3756971

[22] Liang KH, Tso HC, Hung SH, Kuan II, Lai JK, Ke FY, et al. Extracellular domain of EpCAM enhances tumor progression through EGFR signaling in colon cancer cells. Cancer Lett. 2018;433:165–75. 29981429 10.1016/j.canlet.2018.06.040

[23] Pan M, Schinke H, Luxenburger E, Kranz G, Shakhtour J, Libl D, et al. EpCAM ectodomain EpEX is a ligand of EGFR that counteracts EGF-mediated epithelial-mesenchymal transition through modulation of phospho-ERK1/2 in head and neck cancers. PLoS Biol. 2018;16:e2006624. 30261040 10.1371/journal.pbio.2006624 PMC6177200

[24] Schinke H, Shi E, Lin Z, Quadt T, Kranz G, Zhou J, et al. A transcriptomic map of EGFR-induced epithelial-to-mesenchymal transition identifies prognostic and therapeutic targets for head and neck cancer. Mol Cancer. 2022;21:178. 36076232 10.1186/s12943-022-01646-1 PMC9454230

[25] Pan M, Kohlbauer V, Soares AB, Schinke H, Huang YC, Kranz G, et al. Interactome analysis reveals endocytosis and membrane recycling of EpCAM during differentiation of embryonic stem cells and carcinoma cells. Iscience. 2021;24:103179. 10.1016/j.isci.2021.103179 PMC8517208 34693227

[26] Gires O, Pan M, Schinke H, Canis M, Baeuerle PA. Expression and function of epithelial cell adhesion molecule EpCAM: where are we after 40 years? Cancer Metastasis Rev. 2020;39:969–87. 32507912 10.1007/s10555-020-09898-3 PMC7497325

[27] Ying QL, Wray J, Nichols J, Batlle-Morera L, Doble B, Woodgett J, et al. The ground state of embryonic stem cell self-renewal. Nature. 2008;453:519–23. 18497825 10.1038/nature06968 PMC5328678

[28] Zhan T, Ambrosi G, Wandmacher AM, Rauscher B, Betge J, Rindtorff N, et al. MEK inhibitors activate Wnt signalling and induce stem cell plasticity in colorectal cancer. Nat Commun. 2019;10:2197. 31097693 10.1038/s41467-019-09898-0 PMC6522484

[29] Mohammed H, Hernando-Herraez I, Savino A, Scialdone A, Macaulay I, Mulas C, et al. Single-Cell Landscape of Transcriptional Heterogeneity and Cell Fate Decisions during Mouse Early Gastrulation. Cell Rep. 2017;20:1215–28. 28768204 10.1016/j.celrep.2017.07.009 PMC5554778

[30] Pijuan-Sala B, Griffiths JA, Guibentif C, Hiscock TW, Jawaid W, Calero-Nieto FJ, et al. A single-cell molecular map of mouse gastrulation and early organogenesis. Nature. 2019;566:490–5. 30787436 10.1038/s41586-019-0933-9 PMC6522369

[31] Zeng B, Liu Z, Lu Y, Zhong S, Qin S, Huang L, et al. The single-cell and spatial transcriptional landscape of human gastrulation and early brain development. Cell Stem Cell. 2023;30:851–66.e7. 37192616 10.1016/j.stem.2023.04.016 PMC10241223

[32] Haghverdi L, Buttner M, Wolf FA, Buettner F, Theis FJ. Diffusion pseudotime robustly reconstructs lineage branching. Nat Methods. 2016;13:845–8. 27571553 10.1038/nmeth.3971

[33] Li Q. scTour: a deep learning architecture for robust inference and accurate prediction of cellular dynamics. Genome Biol. 2023;24:149. 37353848 10.1186/s13059-023-02988-9 PMC10290357

[34] Holtzinger A, Rosenfeld GE, Evans T. Gata4 directs development of cardiac-inducing endoderm from ES cells. Dev Biol. 2010;337:63–73. 19850025 10.1016/j.ydbio.2009.10.003 PMC2799892

[35] Schroder CM, Zissel L, Mersiowsky SL, Tekman M, Probst S, Schule KM, et al. EOMES establishes mesoderm and endoderm differentiation potential through SWI/SNF-mediated global enhancer remodeling. Dev Cell. 2025;60:735–48.e5. 39662466 10.1016/j.devcel.2024.11.014

[36] Evseenko D, Zhu Y, Schenke-Layland K, Kuo J, Latour B, Ge S, et al. Mapping the first stages of mesoderm commitment during differentiation of human embryonic stem cells. Proc Natl Acad Sci USA. 2010;107:13742–7. 10.1073/pnas.1002077107 PMC2922221 20643952

[37] Kim WT, Choi HS, Lee HM, Jang YJ, Ryu CJ B-cell receptor associated protein 31 regulates human embryonic stem cell adhesion, stemness, and survival via control of epithelial cell adhesion molecule. Stem Cells. 2014. 10.1002/stem.1765 24898727

[38] Yu T, Ma Y, Wang H. EpCAM Intracellular Domain Promotes Porcine Cell Reprogramming by Upregulation of Pluripotent Gene Expression via Beta-catenin Signaling. Sci Rep. 2017;7:46315. 28393933 10.1038/srep46315 PMC5385527

[39] Xiao D, Xiong M, Wang X, Lyu M, Sun H, Cui Y, et al. Regulation of the Function and Expression of EpCAM. Biomedicines. 2024;107:13742–7. 10.3390/biomedicines12051129 PMC11117676 38791091

[40] Schnell U, Cirulli V, Giepmans BN. EpCAM: structure and function in health and disease. Biochim Biophys Acta. 2013;1828:1989–2001. 23618806 10.1016/j.bbamem.2013.04.018

[41] Schnell U, Kuipers J, Giepmans BN. EpCAM proteolysis: new fragments with distinct functions? Biosci Rep. 2013;33:e00030. 23409978 10.1042/BSR20120128 PMC3601740

[42] Slanchev K, Carney TJ, Stemmler MP, Koschorz B, Amsterdam A, Schwarz H, et al. The epithelial cell adhesion molecule EpCAM is required for epithelial morphogenesis and integrity during zebrafish epiboly and skin development. PLoS Genet. 2009;5:e1000563. 19609345 10.1371/journal.pgen.1000563 PMC2700972

[43] Maghzal N, Vogt E, Reintsch W, Fraser JS, Fagotto F. The tumor-associated EpCAM regulates morphogenetic movements through intracellular signaling. J Cell Biol. 2010;191:645–59. 20974811 10.1083/jcb.201004074 PMC3003323

[44] Mitsui K, Tokuzawa Y, Itoh H, Segawa K, Murakami M, Takahashi K, et al. The homeoprotein Nanog is required for maintenance of pluripotency in mouse epiblast and ES cells. Cell. 2003;113:631–42. 12787504 10.1016/s0092-8674(03)00393-3

[45] Torres J, Watt FM. Nanog maintains pluripotency of mouse embryonic stem cells by inhibiting NFkappaB and cooperating with Stat3. Nat Cell Biol. 2008;10:194–201. 18223644 10.1038/ncb1680

[46] Do DV, Ueda J, Messerschmidt DM, Lorthongpanich C, Zhou Y, Feng B, et al. A genetic and developmental pathway from STAT3 to the OCT4-NANOG circuit is essential for maintenance of ICM lineages in vivo. Genes Dev. 2013;27:1378–90. 23788624 10.1101/gad.221176.113 PMC3701193

[47] Lu H, Ma J, Yang Y, Shi W, Luo L. EpCAM is an endoderm-specific Wnt derepressor that licenses hepatic development. Dev Cell. 2013;24:543–53. 23484855 10.1016/j.devcel.2013.01.021

[48] Causeret F, Sumia I, Pierani A. Kremen1 and Dickkopf1 control cell survival in a Wnt-independent manner. Cell Death Differ. 2016;23:323–32. 26206087 10.1038/cdd.2015.100 PMC4716294

[49] Zhang H, Fraser ST, Papazoglu C, Hoatlin ME, Baron MH. Transcriptional activation by the Mixl1 homeodomain protein in differentiating mouse embryonic stem cells. Stem Cells. 2009;27:2884–95. 19711456 10.1002/stem.203 PMC3562714

[50] Kokity L, Czimmerer Z, Benyhe-Kis B, Poscher A, Belai E, Steinbach G, et al. Brachyury co-operates with polycomb protein RYBP to regulate gastrulation and axial elongation in vitro. Front Cell Dev Biol. 2024;12:1498346. 39676794 10.3389/fcell.2024.1498346 PMC11638158

[51] Pfeiffer MJ, Quaranta R, Piccini I, Fell J, Rao J, Ropke A, et al. Cardiogenic programming of human pluripotent stem cells by dose-controlled activation of EOMES. Nat Commun. 2018;9:440. 29382828 10.1038/s41467-017-02812-6 PMC5789885

[52] Russ AP, Wattler S, Colledge WH, Aparicio SA, Carlton MB, Pearce JJ, et al. Eomesodermin is required for mouse trophoblast development and mesoderm formation. Nature. 2000;404:95–9. 10716450 10.1038/35003601

[53] van den Ameele J, Tiberi L, Bondue A, Paulissen C, Herpoel A, Iacovino M, et al. Eomesodermin induces Mesp1 expression and cardiac differentiation from embryonic stem cells in the absence of Activin. EMBO Rep. 2012;13:355–62. 22402664 10.1038/embor.2012.23 PMC3321156

[54] Bardot E, Calderon D, Santoriello F, Han S, Cheung K, Jadhav B, et al. Foxa2 identifies a cardiac progenitor population with ventricular differentiation potential. Nat Commun. 2017;8:14428. 28195173 10.1038/ncomms14428 PMC5316866

[55] Scheibner K, Schirge S, Burtscher I, Buttner M, Sterr M, Yang D, et al. Epithelial cell plasticity drives endoderm formation during gastrulation. Nat Cell Biol. 2021;23:692–703. 34168324 10.1038/s41556-021-00694-x PMC8277579

[56] David R, Jarsch VB, Schwarz F, Nathan P, Gegg M, Lickert H, et al. Induction of MesP1 by Brachyury(T) generates the common multipotent cardiovascular stem cell. Cardiovasc Res. 2011;92:115–22. 21632880 10.1093/cvr/cvr158

[57] Liu Y, Schwartz RJ. Transient Mesp1 expression: a driver of cardiac cell fate determination. Transcription. 2013;4:92–6. 23584093 10.4161/trns.24588 PMC4042590

[58] Costello I, Pimeisl IM, Drager S, Bikoff EK, Robertson EJ, Arnold SJ. The T-box transcription factor Eomesodermin acts upstream of Mesp1 to specify cardiac mesoderm during mouse gastrulation. Nat Cell Biol. 2011;13:1084–91. 21822279 10.1038/ncb2304 PMC4531310

[59] Grigorash BB, van Essen D, Liang G, Grosse L, Emelyanov A, Kang Z, et al. p16(High) senescence restricts cellular plasticity during somatic cell reprogramming. Nat Cell Biol. 2023;25:1265–78. 37652981 10.1038/s41556-023-01214-9

[60] Li H, Collado M, Villasante A, Strati K, Ortega S, Canamero M, et al. The Ink4/Arf locus is a barrier for iPS cell reprogramming. Nature. 2009;460:1136–9. 19668188 10.1038/nature08290 PMC3578184

[61] Rao J, Pfeiffer MJ, Frank S, Adachi K, Piccini I, Quaranta R, et al. Stepwise Clearance of Repressive Roadblocks Drives Cardiac Induction in Human ESCs. Cell Stem Cell. 2016;18:341–53. 26748419 10.1016/j.stem.2015.11.019

[62] Lin CW, Liao MY, Lin WW, Wang YP, Lu TY, Wu HC. Epithelial cell adhesion molecule regulates tumor initiation and tumorigenesis via activating reprogramming factors and epithelial-mesenchymal transition gene expression in colon cancer. J Biol Chem. 2012;287:39449–59. 22989882 10.1074/jbc.M112.386235 PMC3501065

[63] Sankpal NV, Fleming TP, Sharma PK, Wiedner HJ, Gillanders WE. A double-negative feedback loop between EpCAM and ERK contributes to the regulation of epithelial-mesenchymal transition in cancer. Oncogene. 2017;36:3706–17. 10.1038/onc.2016.504 PMC5571977 28192403

[64] Horns F, Martinez JA, Fan C, Haque M, Linton JM, Tobin V, et al. Engineering RNA export for measurement and manipulation of living cells. Cell. 2023;186:3642–58.e32. 37437570 10.1016/j.cell.2023.06.013 PMC10528933

[65] Love MI, Huber W, Anders S. Moderated estimation of fold change and dispersion for RNA-seq data with DESeq2. Genome Biol. 2014;15:550. 25516281 10.1186/s13059-014-0550-8 PMC4302049

[66] Hao Y, Hao S, Andersen-Nissen E, Mauck WM 3rd, Zheng S, et al. Integrated analysis of multimodal single-cell data. Cell. 2021;184:3573–87.e29. 34062119 10.1016/j.cell.2021.04.048 PMC8238499

[67] Wolf FA, Angerer P, Theis FJ. SCANPY: large-scale single-cell gene expression data analysis. Genome Biol. 2018;19:15. 29409532 10.1186/s13059-017-1382-0 PMC5802054

[68] Xu Y, Zhang T, Zhou Q, Hu M, Qi Y, Xue Y, et al. A single-cell transcriptome atlas profiles early organogenesis in human embryos. Nat Cell Biol. 2023;25:604–15. 36928764 10.1038/s41556-023-01108-w

[69] Schubert M, Klinger B, Klunemann M, Sieber A, Uhlitz F, Sauer S, et al. Perturbation-response genes reveal signaling footprints in cancer gene expression. Nat Commun. 2018;9:20. 29295995 10.1038/s41467-017-02391-6 PMC5750219

