# Supplementary material for: EpCAM supports exit from pluripotency of embryonic stem cells via Eomes
Source: Cell Death Dis. 2026 Apr 11;17(1):389. doi: 10.1038/s41419-026-08734-w (PMC13076738; doi:10.1038/s41419-026-08734-w)
Supplement: Supplementary file 1 — Original Data [file 41419_2026_8734_MOESM1_ESM.pdf]

Immunoblots Figure 1D

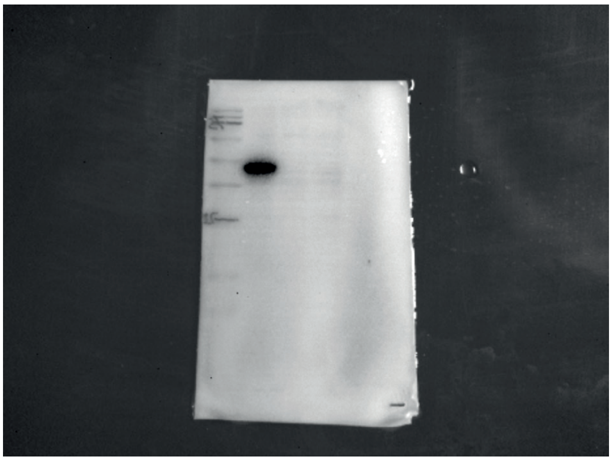
anti-EpCAM

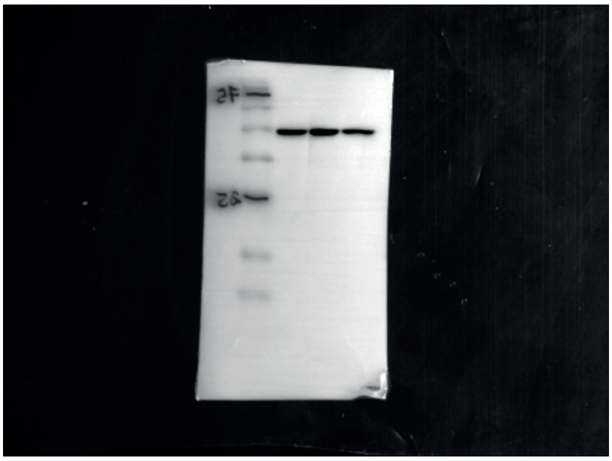
anti-Actin

Immunoblots Suppl. Figure 3A EpCAM-knockout clone #56

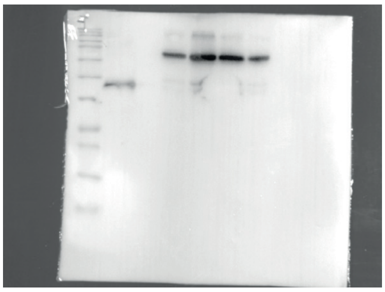
anti-EpCAM

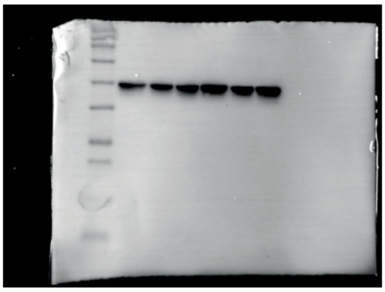
anti-Actin

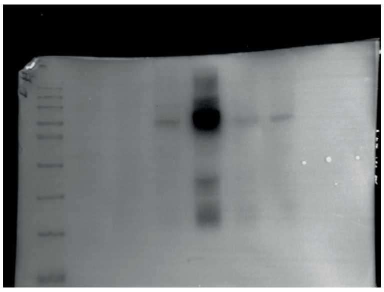
anti-Eomes

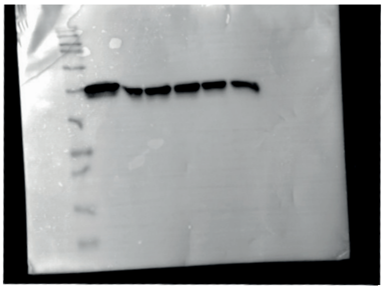
anti-Actin

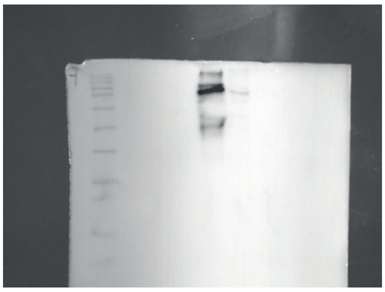
anti-Foxa2

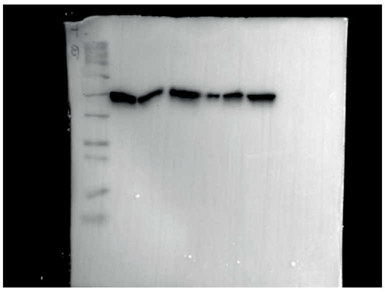
anti-Actin

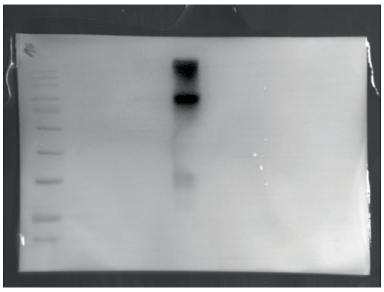
anti-Gata6

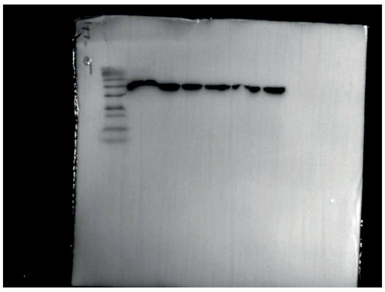
anti-Actin

Immunoblots Suppl. Figure 3A EpCAM-knockout clone #114

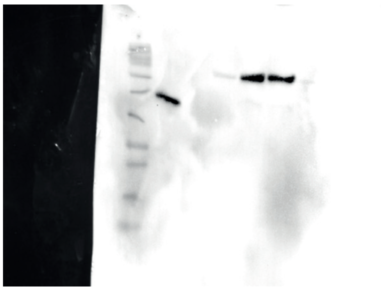
anti-EpCAM

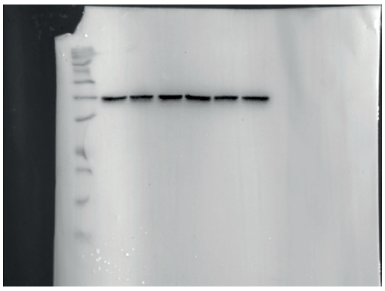
anti-Actin

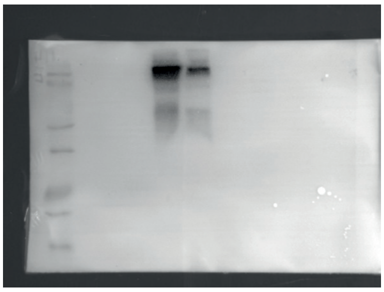
anti-Eomes

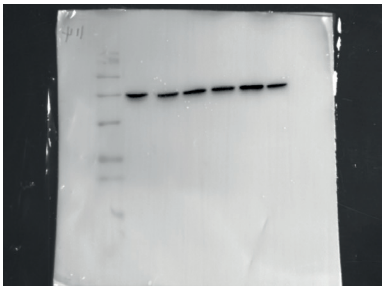
anti-Actin

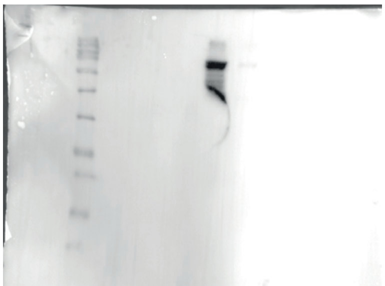
anti-Foxa2

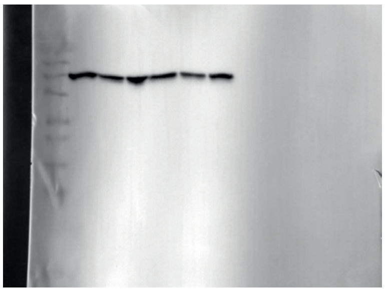
anti-Actin

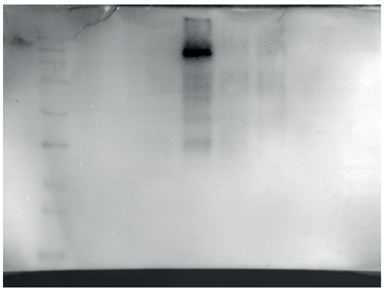
anti-Gata6

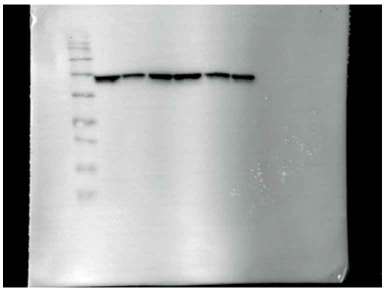
anti-Actin
