# Supplementary material for: EpCAM supports exit from pluripotency of embryonic stem cells via Eomes
Source: Cell Death Dis. 2026 Apr 11;17(1):389. doi: 10.1038/s41419-026-08734-w (PMC13076738; doi:10.1038/s41419-026-08734-w)
Supplement: Supplementary file 2 — Supplementary Figures [file 41419_2026_8734_MOESM2_ESM.pdf]

Supplementary figures for:

EpCAM Supports Exit from Pluripotency of Embryonic Stem Cells *via Eomes*

Ningyue Gong[1,#], Mahesh Gouda[1,#], Ana Marija Balaz[1], Jiahang Song[1], Gisela Kranz[1], Julia Hess[2,3], Philipp Baumeister[1], Kristian Unger[3,4, 5, 6, 2], Vera Katalina [1], Martin Canis[1,§], and Olivier Gires[1, §]

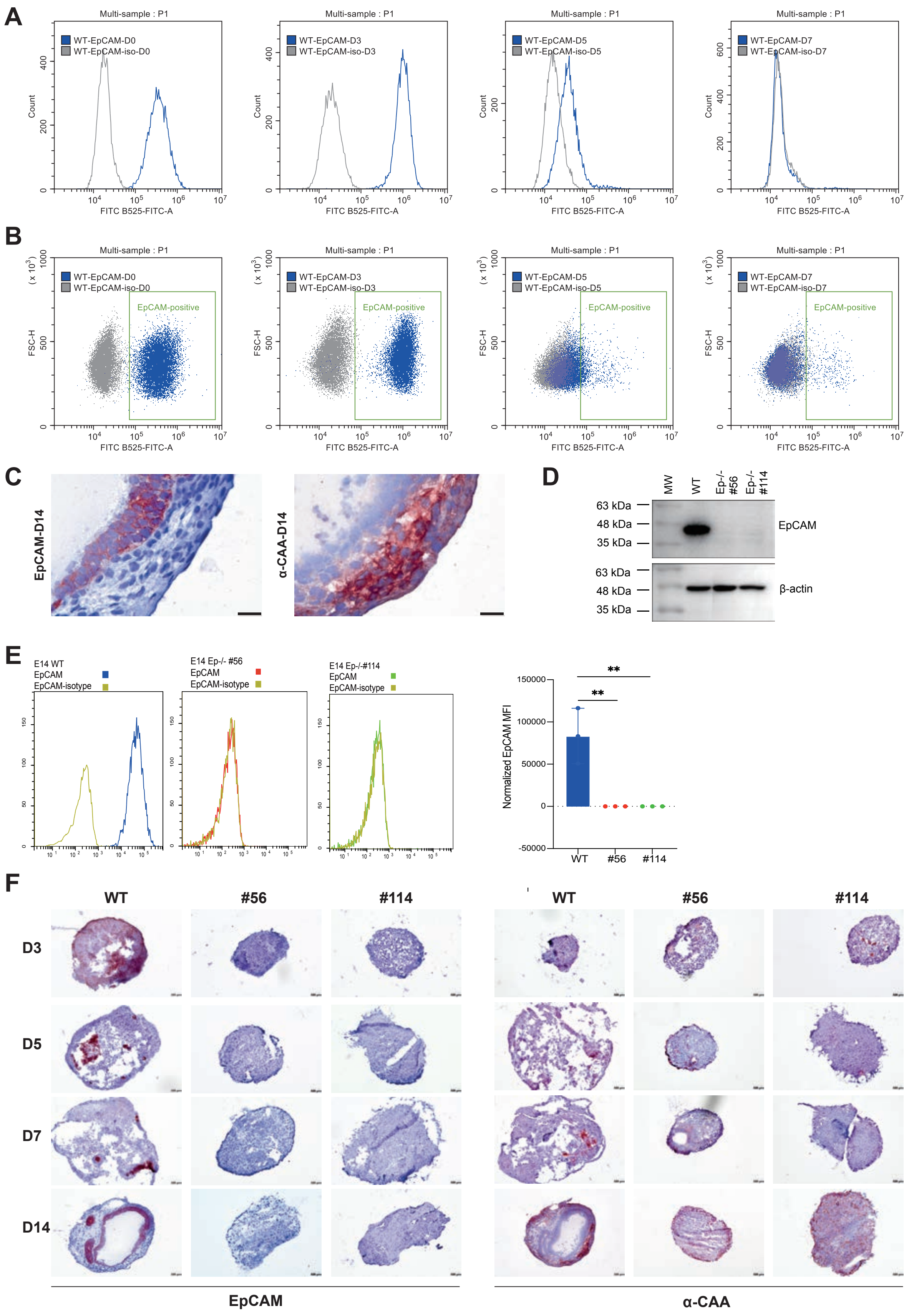

Supplementary Figure 1: EpCAM expression dynamics and knockout in ESCs. (**A-B**) EpCAM expression was analyzed in WT E14TG2α cells during spontaneous differentiation at days 0, 3, 5, and 7 by flow cytometry. (A) Representative histograms, (B) Representative dot plots. (**C**) Immunohistochemical staining of EpCAM and α-CAA in EBs of WT E14TG2α during spontaneous differentiation at day 14. Shown are representative staining from n = 3 independent experiments. (**D**)

EpCAM expression was analyzed in whole cell lysates of WT and EpCAM$^{-/-}$ cells by immunobloting. Shown is one representative experiments from n = 3 independent repeats. (**E**) Cell surface expression of EpCAM in WT and EpCAM$^{-/-}$ E14TG2α was analyzed by flow cytometry. Shown are representative histograms with isotype controls and normalized MFIs from n = 3 independent experiments. ** p-value < 0.01. (**F**) Immunohistochemical staining of EpCAM and α-CAA in EBs of WT and EpCAM$^{-/-}$ E14TG2α during spontaneous differentiation at the indicated time points. Shown are representative staining from n = 3 independent experiments.

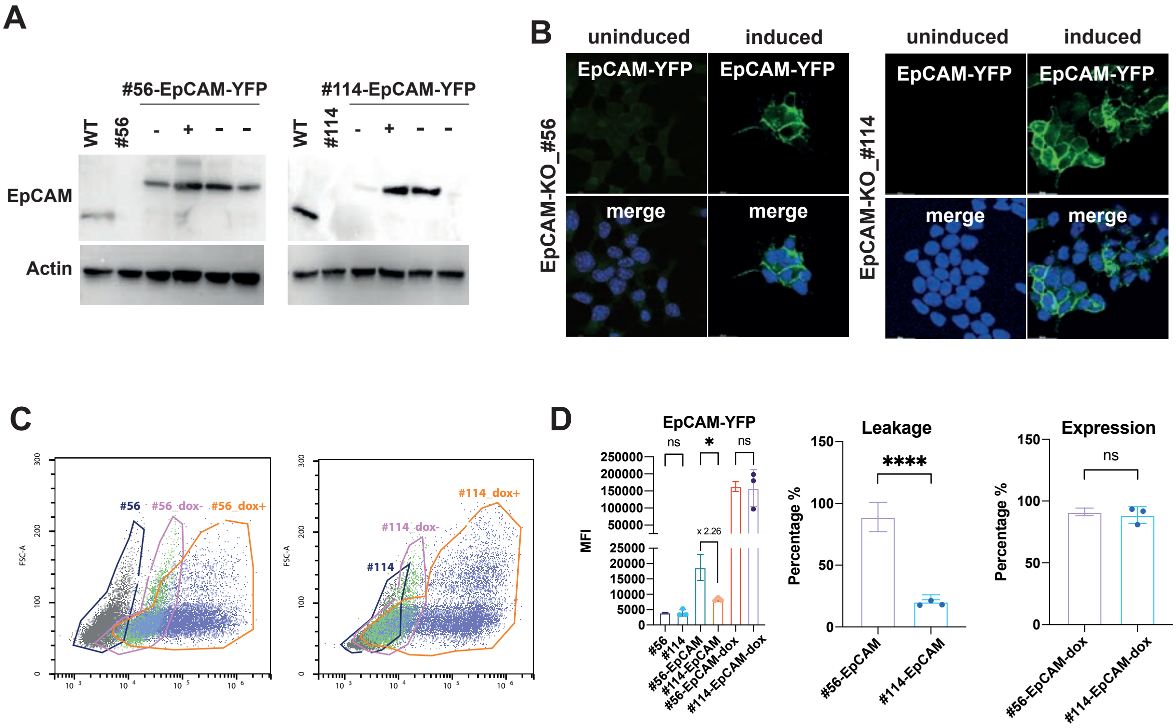

Supplementary Figure 2: Conditional expression of EpCAM-YFP in ESCs. (**A**) E14TG2α EpCAM$^{-/-}$ ESC clone #56 and #114 were stably transfected with the indicated conditional expression plasmid and were treated as follows: untreated (-), doxycycline treatment (24h, +), doxycycline treatment 24h and withdrawal for 24h and 48h (following "-" captures). Shown are representative results from three independent experiments in which wildtype (WT) and untransfected E14TG2α EpCAM$^{-/-}$ ESCs served as negative controls. Comparable protein loading was confirmed by Actin staining. (**B**) Immunofluorescence detection of yellow fluorescence protein (YFP)-tagged EpCAM in E14TG2α EpCAM$^{-/-}$ ESC clone #56 and #114 in confocal, laser-scanning microscopy micrographs. Cellular DNA was detected with DAPI. Shown are representative images from n = 3 independent staining. (**C**) Shown are representative dot-plots of untransfected E14TG2α EpCAM$^{-/-}$ ESC clone #56 and #114, transfected cells in the absence (dox-) and presence (dox+) of doxycycline in the FITC-A channel from n = 3 independent experiments. (**D**) Mean fluorescence intensities (MFI) and percentages of leakage and protein expression are presented as means with SD from n = 3 independent experiments. Leakage refers to the expression of the indicated fusion protein in the absence of doxycycline compared to parental cells. Expression refers to the percentage of fusion protein-positive cells after doxycycline treatment compared to untreated cells.

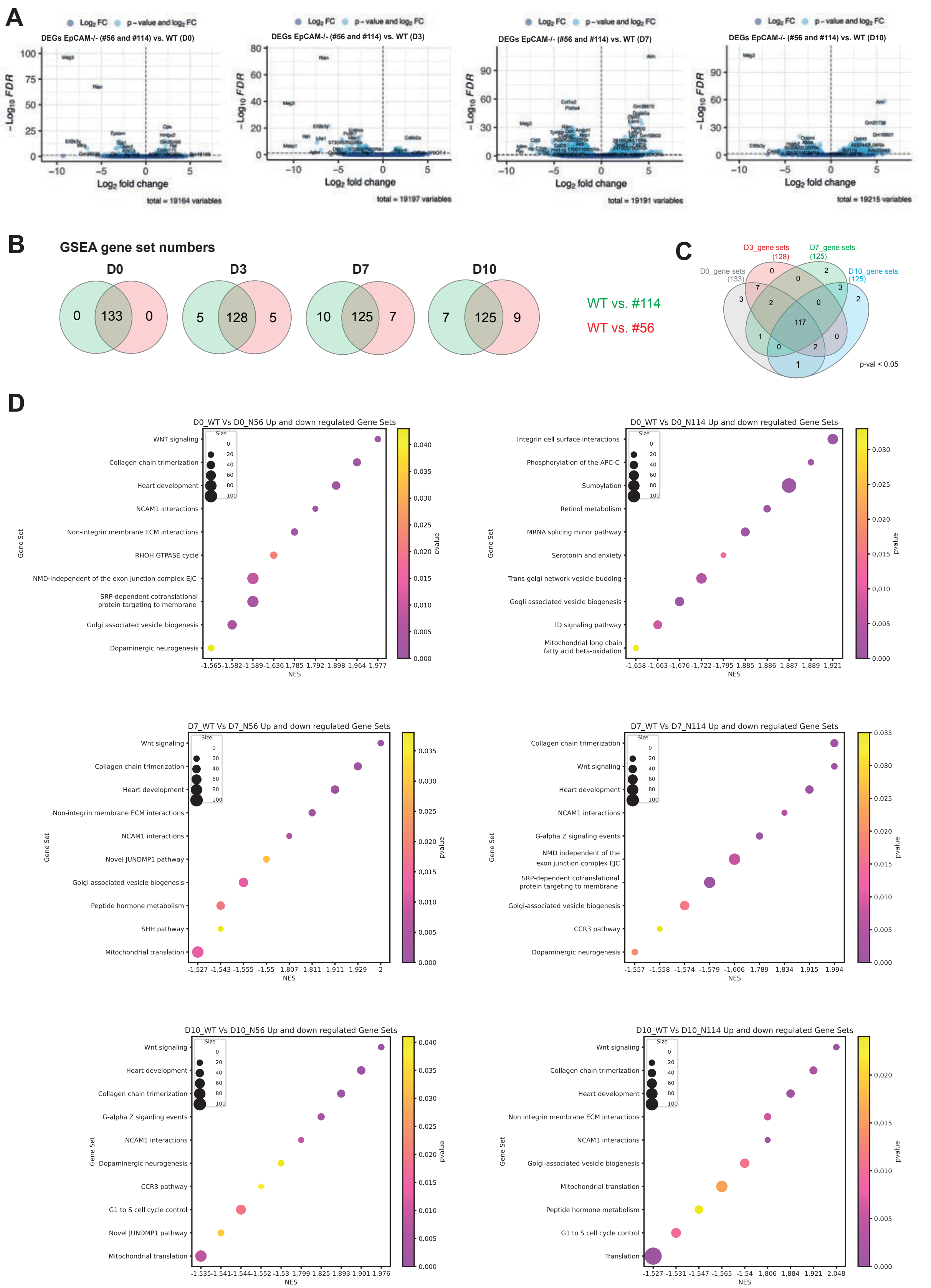

Supplementary Figure 3: EpCAM knockout impacts on hallmarks of heart development and Wnt signaling. (**A**) Volcano plots of differentially expressed genes (DEGs, logFC > 1, p-value < 0.05) between WT and EpCAM-/- cells (clones #56 and #114) are shown for each differentiation day (D0, D3, D7, and D10). (**B**) Venn diagrams of numbers of gene sets (CP within MSigDB) significantly activated or suppressed between WT and EpCAM$^{-/-}$ cells (clones #56 and #114) as determined upon gene set

enrichment analysis (GSEA). (**C**) Venn diagram representing intersections of all significantly changed gene sets between WT versus EpCAM$^{-/-}$ cells (clones #56 and #114). (**D**) Top ten differential gene sets identified upon GSEA using within GSEA-MSigDB are depicted with normalized enrichment scores (NES) and normalized p-values. Shown are the GSEA from WT versus EpCAM$^{-/-}$ cell clones #56 and #114, respectively.

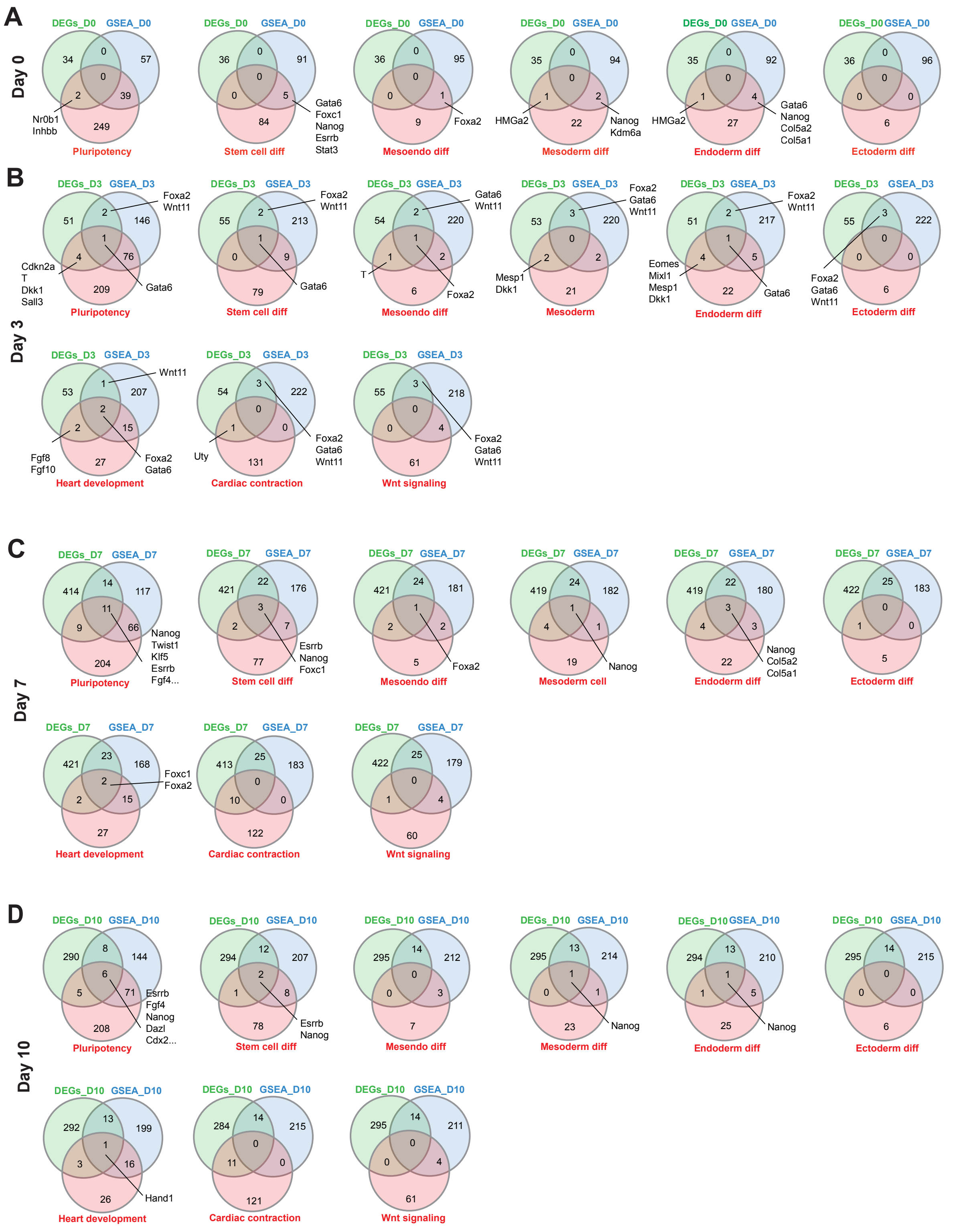

Supplementary Figure 4: Definition of EpCAM-associated functional DEGs in mESC exit from pluripotency. (**A-D**) Venn diagram representation of gene intersections of common differentially expressed genes (DEGs) between WT and EpCAM$^{-/-}$ cells (clones #56 and #114), GSEA analysis, and selected hallmark gene sets as indicated (red fon) at D0 (A), D3 (B), D7 (C), D10 (D). Singular genes are marked at the different time points.

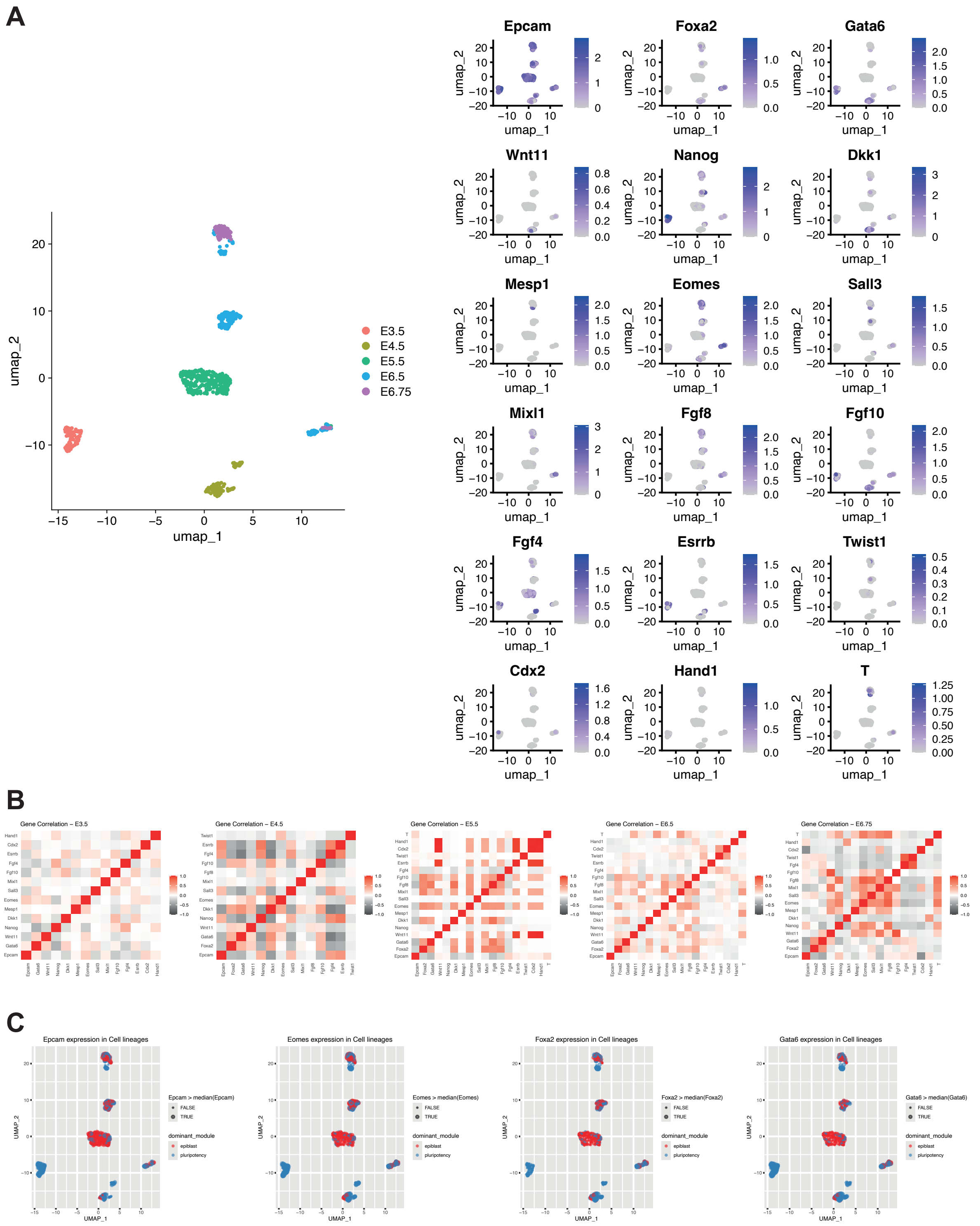

Supplementary Figure 5: Expression of EpCAM and functional DEGs in early murine embryonic development. (**A**) The expression of *Epcam* and related functional DEGs was analyzed at single cell level in GSE100597. Early gastrulation days E3.5, E4.5, E5.5, E6.5, and E6.75 are depicted in a uniform manifold approximation and projection (UMAP) (center, left). Expression of genes of interest is depicted in corresponding UMAPs (right pannels). (**B**) Correlation heatmaps of *Epcam* and functional DEGs are shown for gestation days E3.5, E4.5, E5.5, E6.5, and 6.75. (**C**) Pluripotent (blue) and epiblast (red) cells are depicted as UMAP for E3.5, E4.5, E5.5, E6.5, and E6.75. The expression of *Epcam*, *Eomes*, *Foxa2*, and *Gata6* is shown as small circles (negative, NEG) and as large circles (positive, POS, > mean).

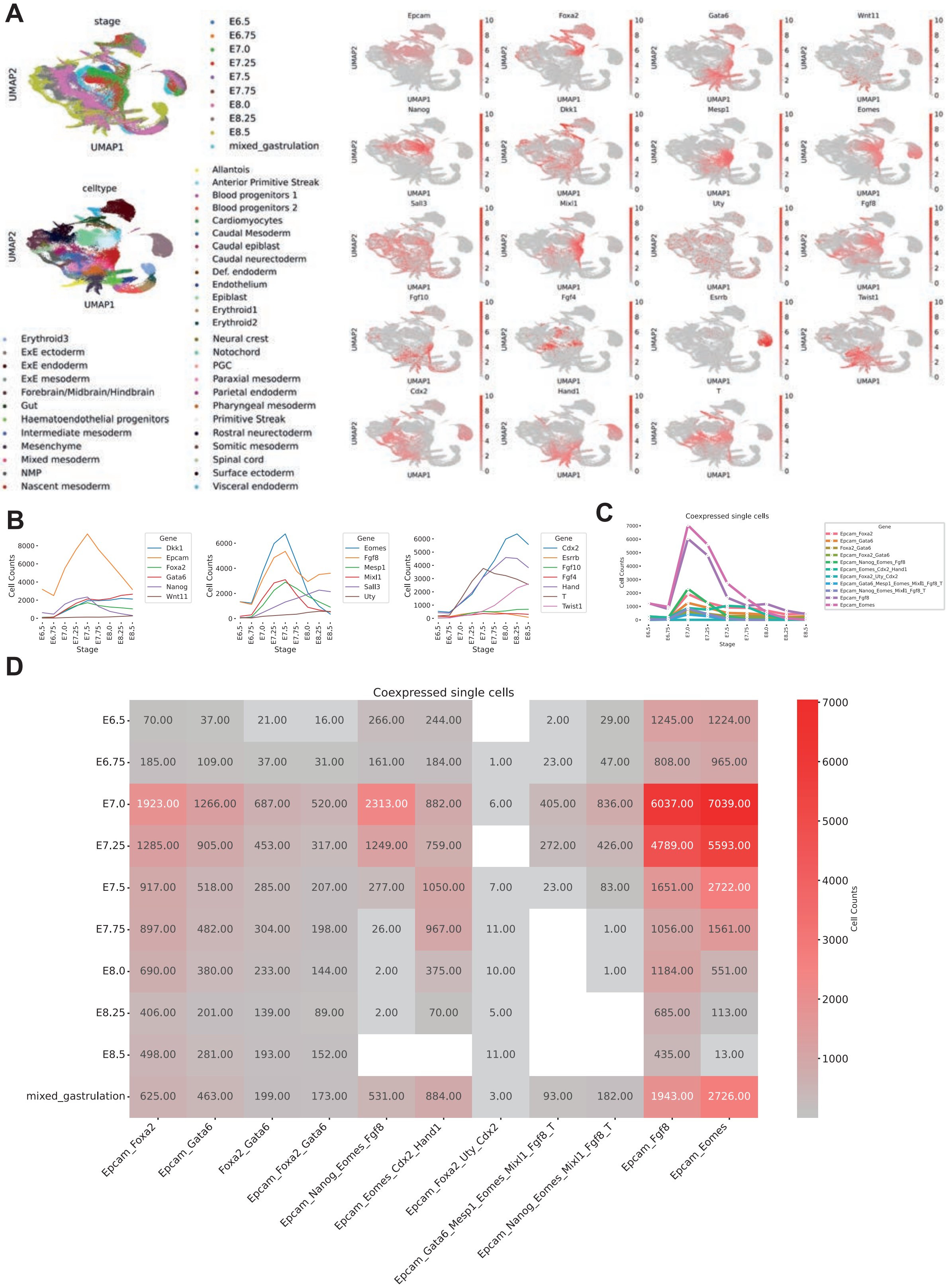

Supplementary Figure 6: *Epcam* and functional DEGs expression in murine embryogenesis. (**A**) UMAP representation of gestation days of murine embryonic development from E6.5 to E8.25 (upper left UMAP), and of lineages and cell types (lower left UMAP). Expression of *Epcam* and n = 18 functional DEGs is depicted at single cell level in individual UMAPs (right panels). (**B-C**) *Epcam* and n = 18 functional DEGs are shown throughout early time points of murine embryonic development as cell numbers for single genes (B) and co-expressions (C). (**D**) Heatmap representation of gene co-expression patterns in single cells throughout early time points of murine embryonic development.

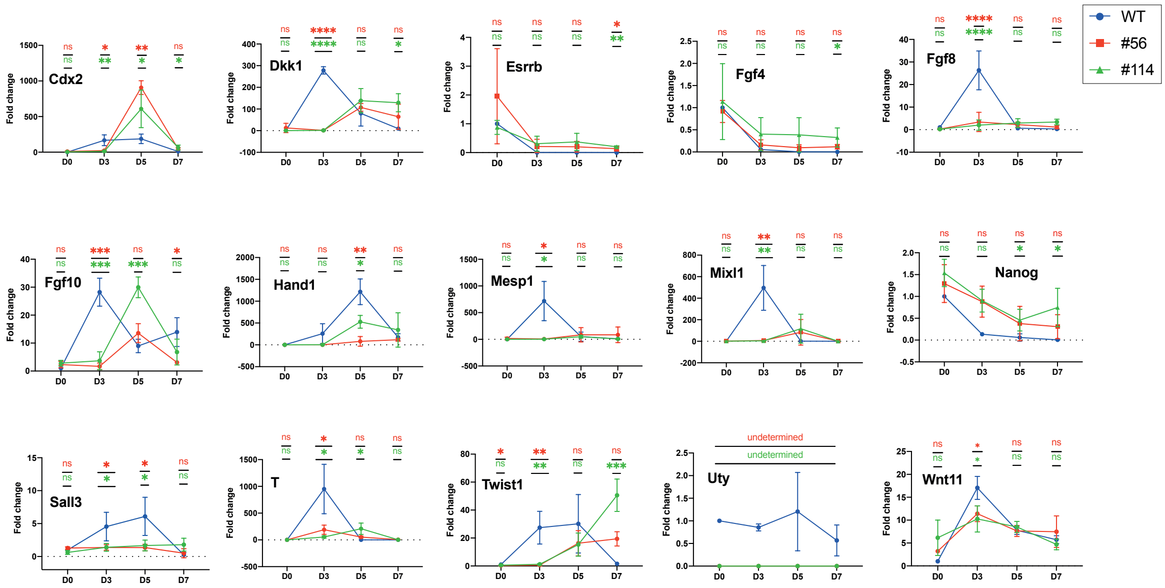

Supplementary Figure 7: Expression of functional DEGs in WT and EpCAM$^{-/-}$. WT and EpCAM$^{-/-}$ ESCs (clones #56 and #114) were subjected to spontaneous differentiation in EBs and were analyzed by qRT-PCR at the indicated time points. Functional DEGs were validated in independent experiments (n = 3). Shown are mean with SD. * p-value < 0.05, ** < 0.01, *** < 0.001, **** < 0.0001; ns: not significant. Expression levels are displayed for WT (blue), EpCAM$^{-/-}$ clones #56 (red) and #114 (green).

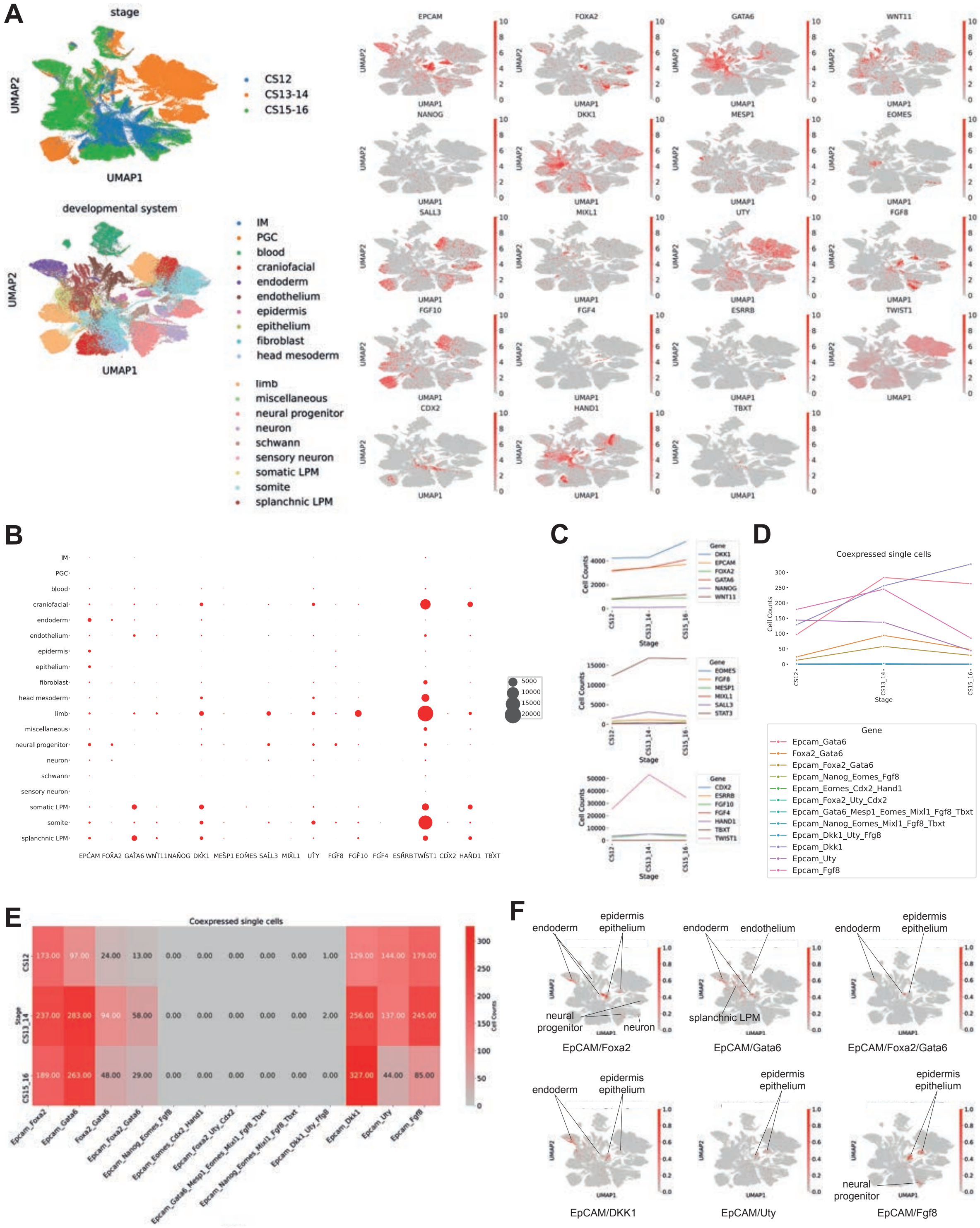

Supplementary Figure 8: *Epcam* and functional DEGs expression in human embryogenesis. (**A**) UMAP representation of Carnegie stages 12-16 of human embryogenesis (upper left UMAP), and of lineages and cell types (lower left UMAP). Expression of *Epcam* and n = 18 functional DEGs is depicted at single cell level in individual UMAPs (right panels). (**B**) Expression of *Epcam* and n = 18 functional DEGs is shown as dot plot graph in the indicated human lineages and cell types. (**C-D**) *Epcam* and n = 18 functional DEGs are shown throughout Carnegie stages of human embryonic development as cell numbers for single genes (C) and gene co-expressions (D). (**E**) Heatmap representation of gene co-expression patterns in single cells throughout early time points of human Carnegie stages. (**F**) UMAP representation of the indicated gene co-expression patterns in lineages of human development.

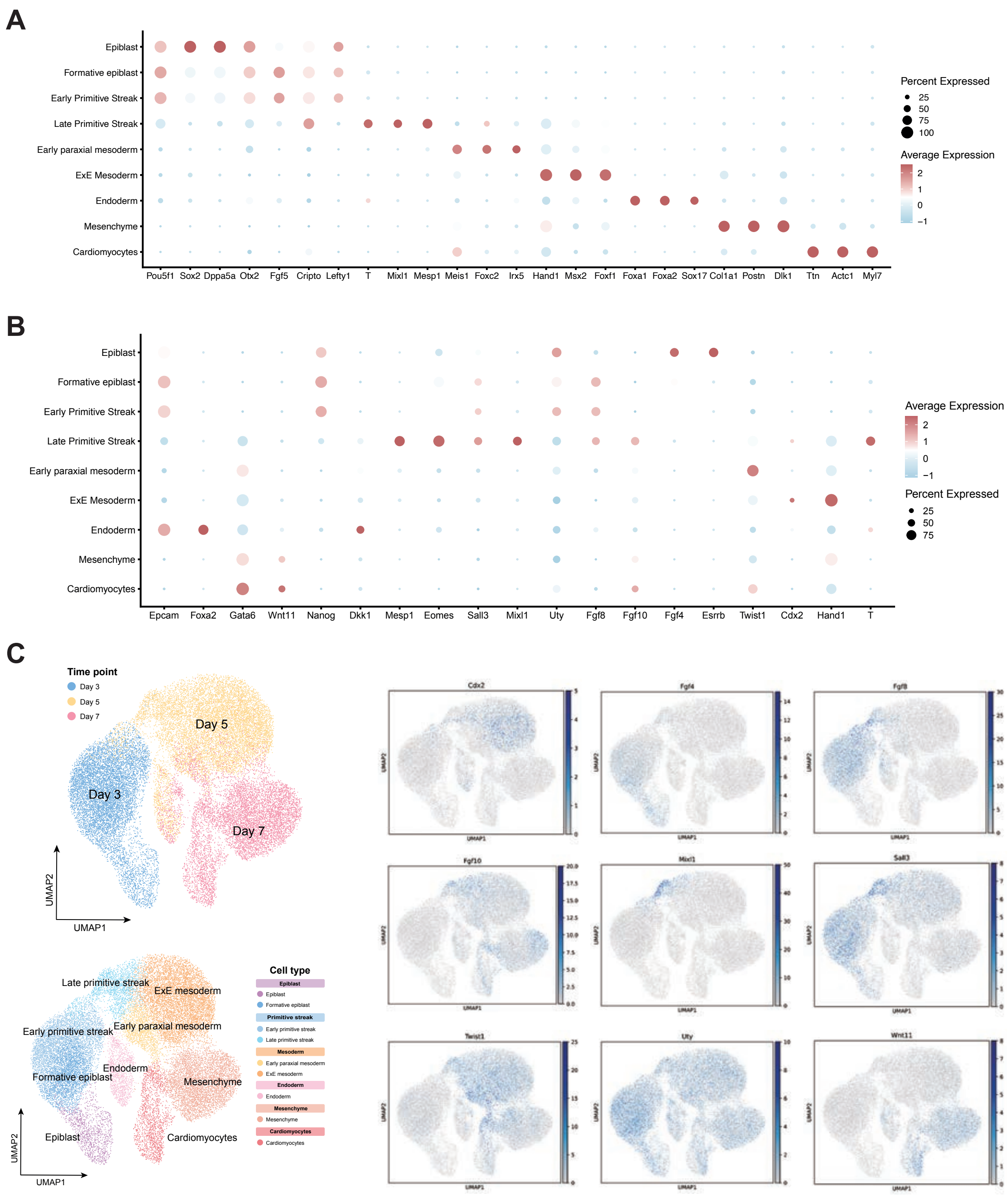

Supplementary Figure 9: EpCAM and fDEGs expression analysis in mESC as single-cell level. (**A**) Expression of marker genes is shown as dot plot graph in the indicated lineages of spontaneously differentiated E14TG2α mESCs. (**B**) Expression of *Epcam* and n = 18 functional DEGs is shown as dot plot graph in the indicated lineages of spontaneously differentiated E14TG2α mESCs. (**C**) UMAP representation of single spontaneously differentiated E14TG2α mESCs at days 3, 5, and 7 (upper left panel) and of annotated cell types as indicated in the legend (lower left panel). The expression of the indicated EpCAM-associated fDGEs is depicted in individual UMAP (right panels).

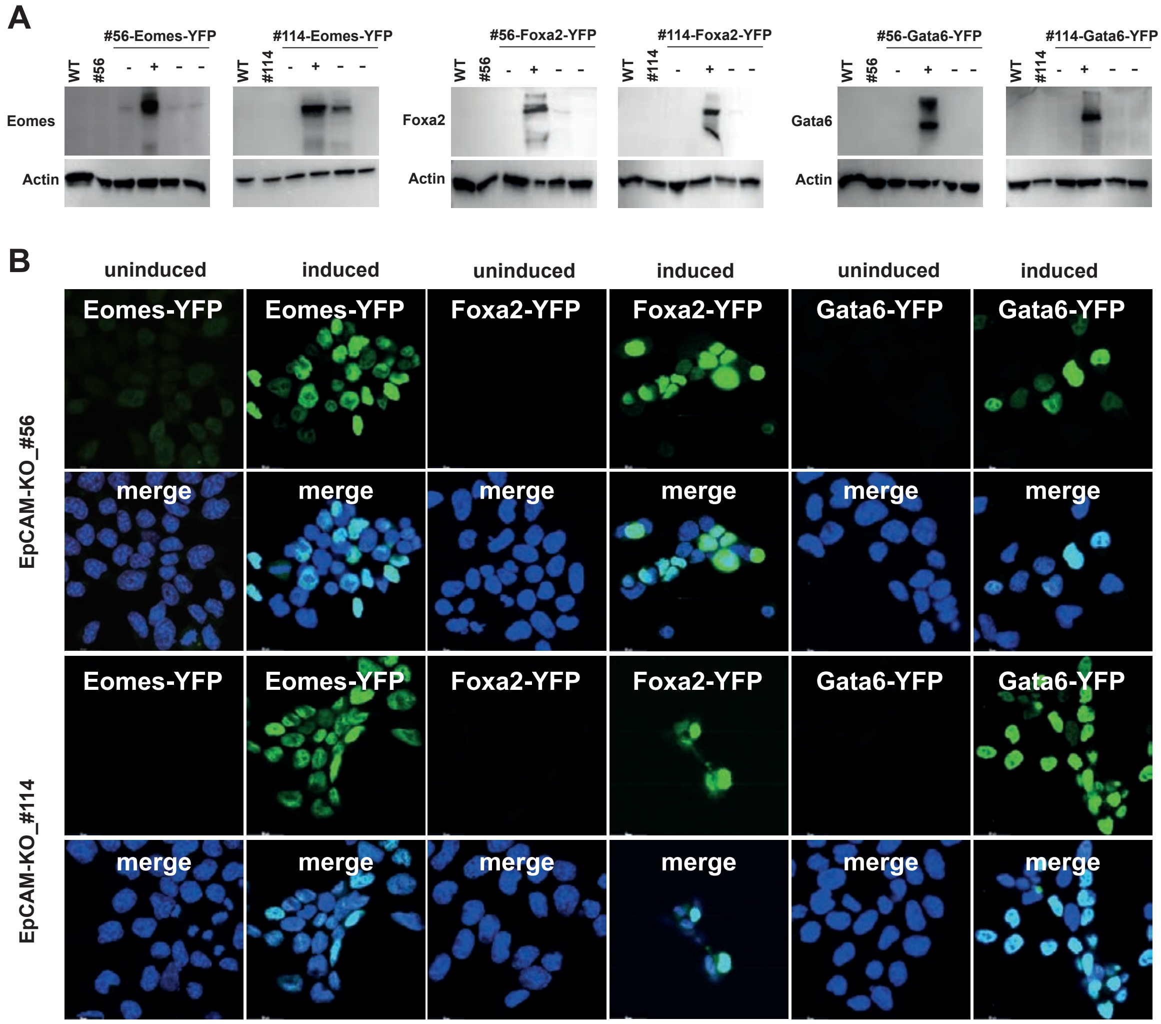

Supplementary Figure 10: Conditional expression of Eomes-YFP, Foxa2-YFP, and Gata6-YFP in ESCs. (**A**) E14TG2α EpCAM$^{-/-}$ ESC clone #56 and #114 were stably transfected with the indicated conditional expression plasmids and were treated as follows: untreated (-), doxycycline treatment (24h, +), doxycycline treatment 24h and withdrawal for 24h and 48h (following "-" captures). Shown are representative results from three independent experiments in which wildtype (WT) and untransfected E14TG2α EpCAM$^{-/-}$ ESCs served as negative controls. Comparable protein loading was confirmed by Actin staining. (**B**) Immunofluorescence detection of yellow fluorescence protein (YFP)-tagged Eomes Foxa2, and Gata6 in E14TG2α EpCAM$^{-/-}$ ESC clone #56 and #114 in confocal, laser-scanning microscopy micrographs. Cellular DNA was detected with DAPI. Shown are representative images from n = 3 independent staining.

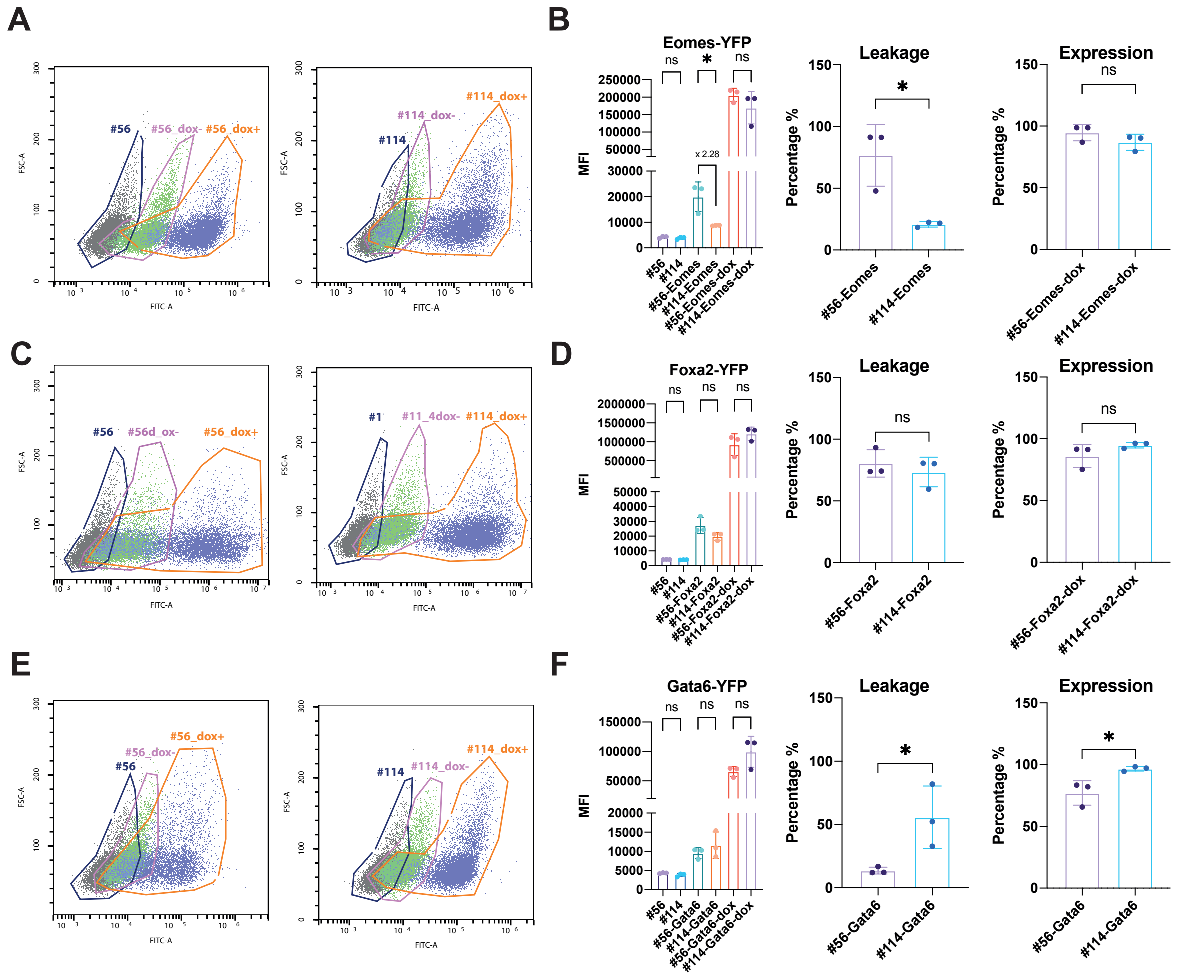

Supplementary Figure 11: Promoter leakage in ESCs. E14TG2α EpCAM$^{-/-}$ ESC clone #56 and #114 were stably transfected with conditional expression plasmids for Eomes-YFP (**A-B**), Foxa2-YFP (**C-D**), and Gata6-YFP (**E-F**). (**A**, **C**, **E**) Shown are representative dot-plots of untransfected E14TG2α EpCAM$^{-/-}$ ESC clone #56 and #114, transfected cells in the absence (dox-) and presence (dox+) of doxycycline in the FITC-A channel from n = 3 independent experiments. (**B**, **D**, **F**) Mean fluorescence intensities (MFI) and percentages of leakage and protein expression are presented as means with SD from n = 3 independent experiments. Leakage refers to the expression of the indicated fusion protein in the absence of doxycycline compared to parental cells. Expression refers to the percentage of fusion protein-positive cells after doxycycline treatment compared to untreated cells.

A

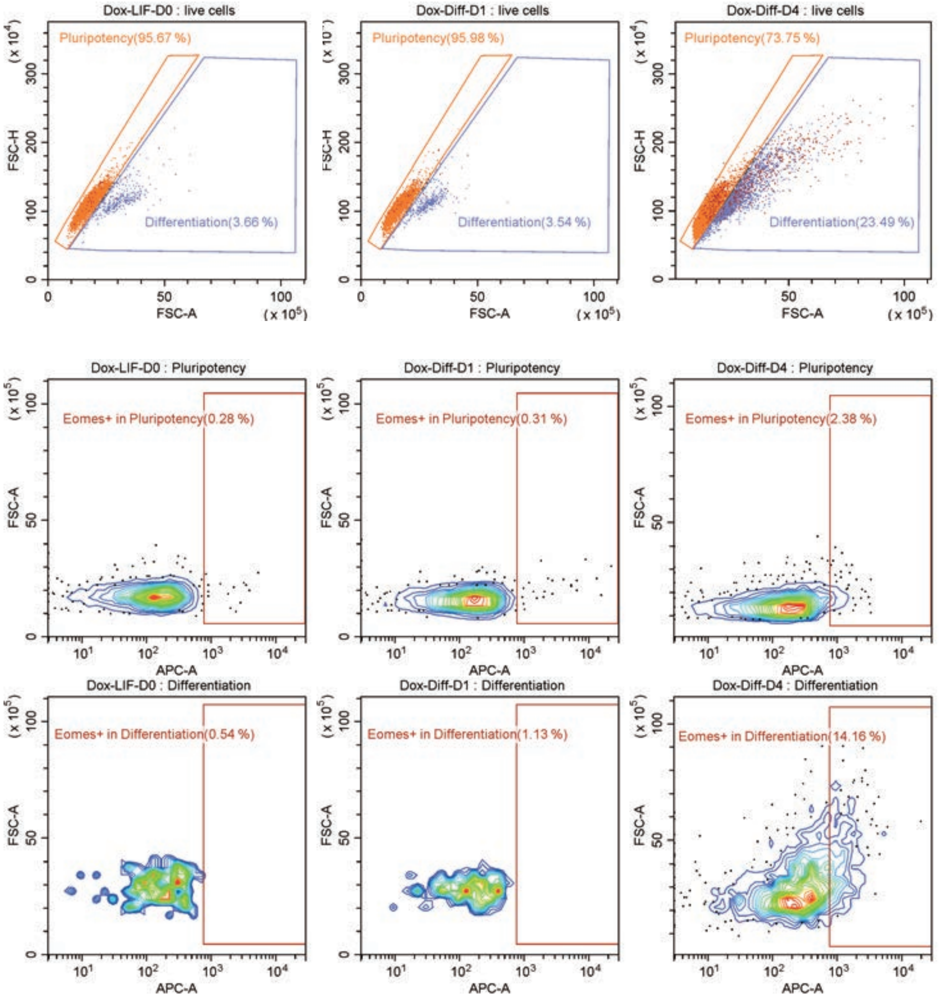

B

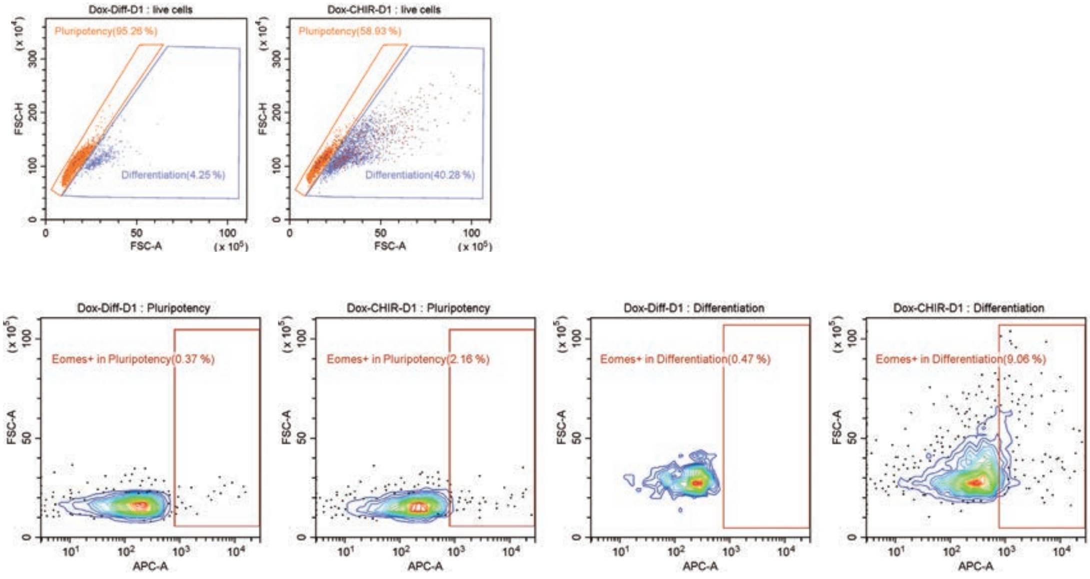

Supplementary Figure 12: Influence of Wnt signaling on differentiation and *Eomes* promoter activity (**A**) E14TG2α EpCAM$^{-/-}$ ESC clone #114 was stably transfected with a conditional *Epcam* expression vector and an *Eomes* promoter-reporter plasmid expressing mCherry fluorescence protein. ESCs were subjected to 2D spontaneous differentiation via LIF withdrawal. E14TG2α EpCAM$^{-/-}$ ESC differentiation was assessed by flow cytometry using forward scatters of area and height (FSC-A vs. FSC-H). Representative dot plots of FSC-A and FSC-H at days 0, 1, and 4 from n = 3 independent experiments are shown. (**B**) E14TG2α EpCAM$^{-/-}$ ESC differentiation in the absence and presence of

CHIR99021 was assessed by flow cytometry using forward scatters of area and height (FSC-A vs. FSC-H). Shown are representative dot plots of FSC-A and FSC-H at day 1 from n = 3 independent experiments. mCherry expression was assessed by flow cytometry in pluripotent and differentiated cells. Shown are representative dot plots from n = 3 independent experiments.

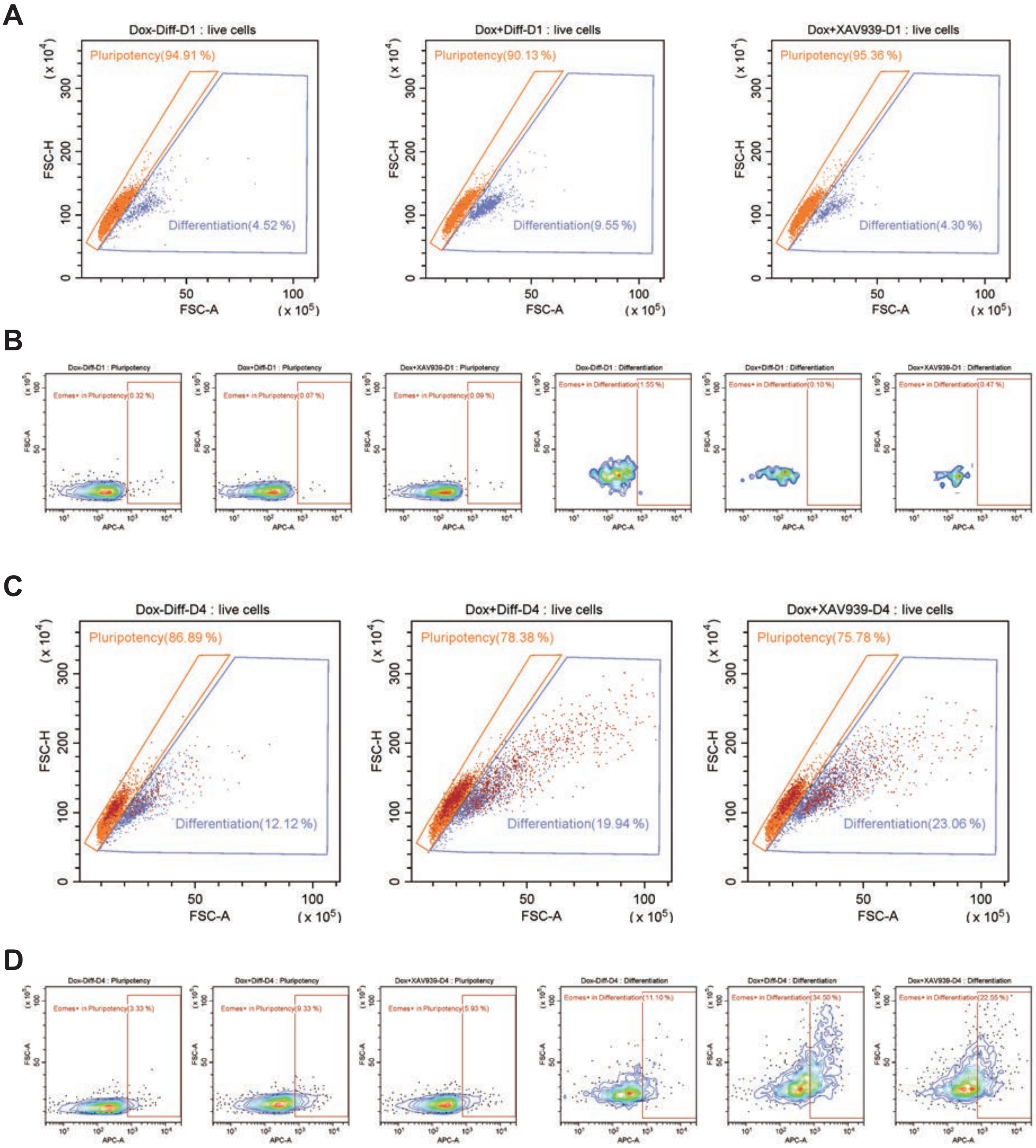

Supplementary Figure 13: EpCAM influence on *Eomes* expression (**A, C**) E14TG2α EpCAM$^{-/-}$ ESC differentiation was assessed by flow cytometry using forward scatters of area and height (FSC-A vs. FSC-H). Shown are representative dot plots of FSC-A and FSC-H at days one (A) and four (C) from n = 3 independent experiments. (**B, D**) mCherry expression, as surrogate marker for *Eomes*-promoter activity, was assessed by flow cytometry in pluripotent and differentiated ESCs. Shown are representative dot plots from n = 3 independent experiments with cells without doxycycline (gray line), with doxycycline for 24 h (light green), and with doxycycline and XAV939 (dark green) at day one (D) and four (F).
