# Supplementary material for: EpCAM supports exit from pluripotency of embryonic stem cells via Eomes
Source: Cell Death Dis. 2026 Apr 11;17(1):389. doi: 10.1038/s41419-026-08734-w (PMC13076738; doi:10.1038/s41419-026-08734-w)
Supplement: Supplementary file 4 — 41419_2026_8734_MOESM4_ESM [file 41419_2026_8734_MOESM4_ESM.docx]

**Supplementary Table 1: Compilation of differentially expressed genes over the course of spontaneous differentiation of mESC.**

Columns A-D: Differentially expressed genes (DEGs, DeSeq) between wildtype E14TG2α mESCs and EpCAM knockout clones #56 and #114 are listed for each individual differentiation time point (D0, 3, 7, and 10).

Columns F-I: Differentially expressed genes (DEGs) between wildtype E14TG2α mESCs and EpCAM knockout clones #56 and #114 were extracted from significantly regulated hallmarks following gene set enrichment analysis (GSEA). Genes are listed for each individual differentiation time point (D0, 3, 7, and 10).
